# Supplementary material for: Chitinase-like protein 3: A novel niche factor for mouse neural stem cells
Source: Stem Cell Reports. 2022 Nov 10;17(12):2704–17. doi: 10.1016/j.stemcr.2022.10.012 (PMC9768575; doi:10.1016/j.stemcr.2022.10.012)
Supplement: Document S1. Figures S1–S7 and supplemental experimental procedures [file mmc1.pdf]

Stem Cell Reports, Volume *17*

Supplemental Information

Chitinase-like protein 3: A novel niche factor for mouse neural stem cells

Jun Namiki, Sayuri Suzuki, Shinsuke Shibata, Yoshiaki Kubota, Naoko Kaneko, Kenji Yoshida, Ryo Yamaguchi, Yumi Matsuzaki, Takeshi Masuda, Yasushi Ishihama, Kazunobu Sawamoto, and Hideyuki Okano

Figure S1

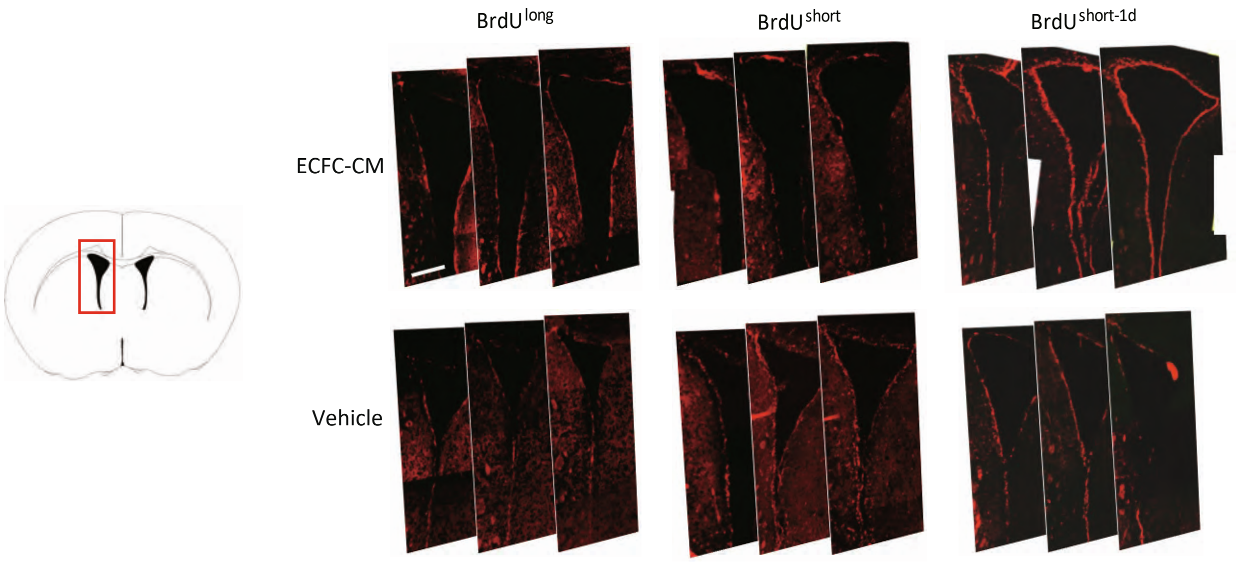

Figure S1. Immunostaining of BrdU-labeled cells (red) in the adult V-SVZ (related to Figure 1). ECFC-CM or vehicle was infused into the lateral ventricle for 7 days and assigned to distinct regimens of BrdU administration: $BrdU^{long}$, $BrdU^{short}$, or $BrdU^{short\text{-}1d}$. Coronal sections were prepared through the anterior part of the lateral ventricles. Representative immunofluorescence images of the ventricular zone (red square) were shown. Scale bar, 200 μm.

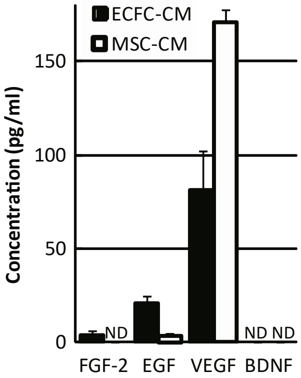

Figure S2. Concentrations of known growth factors in ECFC-CM and MSC-CM measured by ELISA (related to Figures 2B and 2C). ND, not detected. 2 or 3 independent experiments.

Figure S3

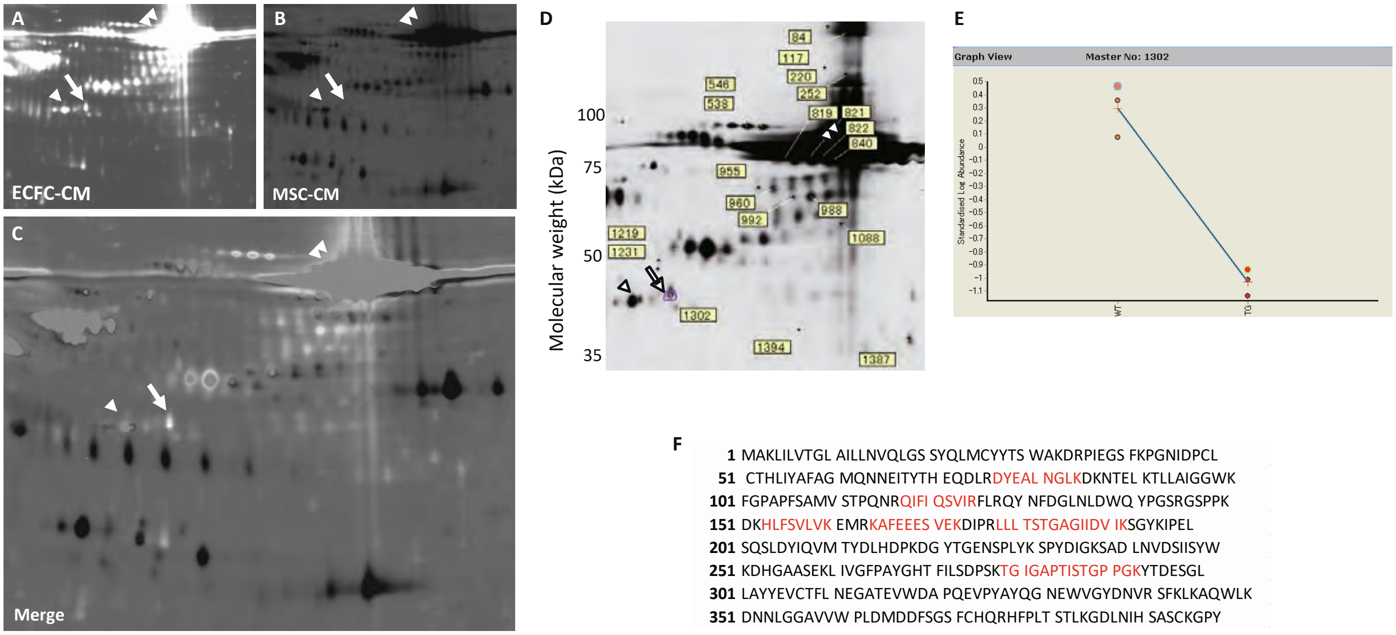

Figure S3. ECFC-CM-specific spots in 2D-DIGE (related to Figures 2D-2G). (A-C) Gray-scale images of 2D-DIGE showing spots from ECFC-CM (white in A and C) and MSC-CM (black in B and C). Arrow, the most ECFC-CM-specific spot; arrowheads and double arrowheads, nonspecific spots of β-actin and transferrin, respectively. (D) Spots of ECFC-CM for which the protein abundance ratio (ECFC-CM/MSC-CM) was > 4 (3 independent experiments, $p < 0.01$). Numbers correspond to Master No. in Figure 2G. (E) The spot most specific to ECFC-CM exposure (No. 1302) showing 22.3-fold more abundant than the corresponding spot after MSC-CM exposure ($p = 0.0005$). (F) Matched peptides of spot 1302 with the protein database search. The eluate of spot 1302 was processed for nanoLC-MS/MS and matched with seven peptides (red) from CHIL3 (sequence coverage = 16% of 398 amino acids, protein score = 370) in Swiss-Prot and NCBInr.

Figure S4

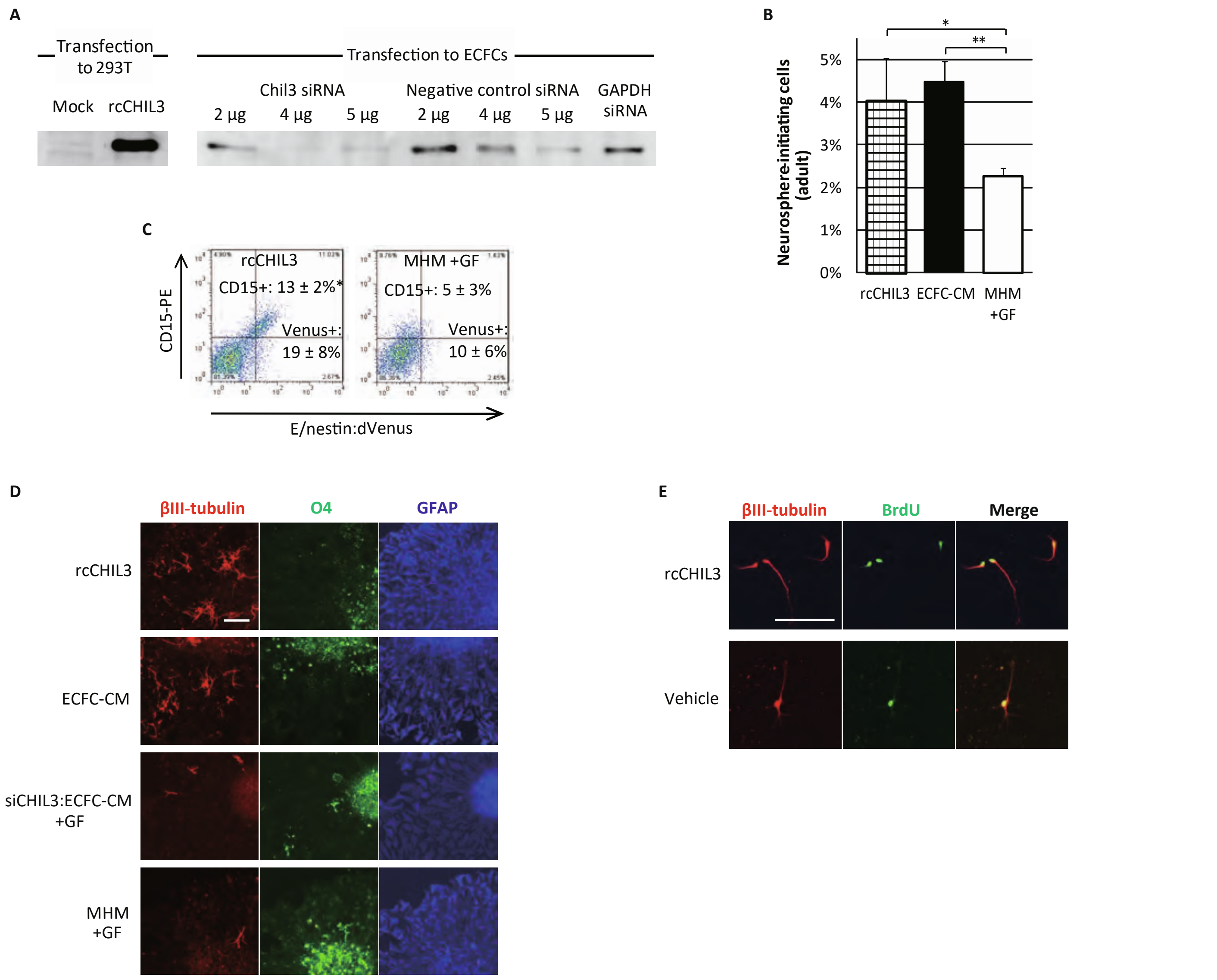

Figure S4. The neurosphere assay, flow cytometry of NSC markers, differentiation of neurospheres, and *ex vivo* differentiation of V-SVZ cells showing that rcCHIL3 promotes NSC self-renewal and neurogenesis (related to Figures 3A-3F). (A) Immunoblotting of rcCHIL3 and *Chil3* siRNA using CHIL3 antibody. Recombinant Venus protein was made as a mock experiment. siRNA that had no homology to any known mammalian gene was used as a negative control. CHIL3 was effectively depleted in ECFC-CM with *Chil3* siRNA of 4 or 5 µg/well. (B) Adult neurosphere assay with rcCHIL3, ECFC-CM, or MHM + GF. Data are mean ± SEM, $*p < 0.05$, $**p < 0.01$. 5 or 10 independent experiments. (C) Plots of flow cytometry showing expression levels of CD15 and Venus in the neurosphere cells treated with rcCHIL3 or MHM + GF. E/nestin:dVenus, a destabilized fluorescent protein Venus under the control of *Nes* second intronic enhancer. $*p < 0.05$, t-test versus MHM + GF controls. (D) Triple labeling immunocytochemistry after differentiation of neurospheres treated with rcCHIL3, ECFC-CM, siCHIL3:ECFC-CM+GF, or MHM + GF. βIII-tubulin (neurons, red), O4 (oligodendrocytes, green), and GFAP (astrocytes, blue). (E) Double labeling images of SVZ NSCs after *ex vivo* differentiation. βIII-tubulin (neurons, red) and BrdU (green). Adult mouse SVZ cells were dissected after 7 days of ventricular infusion with rcCHIL3 or vehicle, and cultured to differentiate with BrdU. Scale bar, 100 µm.

Figure S5

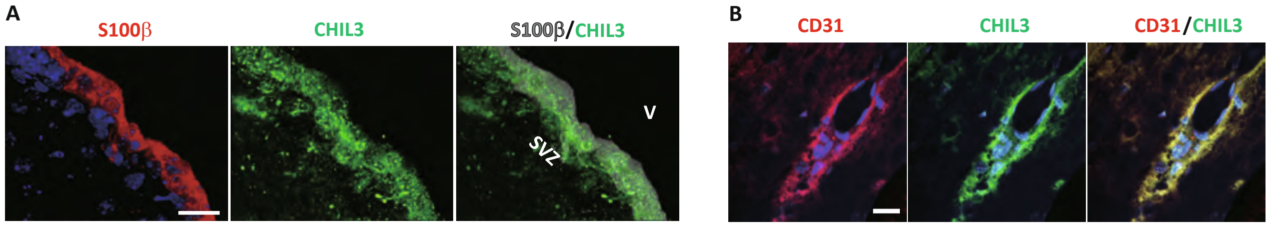

Figure S5. CHIL3 expression of S100β-positive ependymal cells (related to Figure 4J), and vascular cells in the brain after injury (related to Figure 5C). (A) The ventricular ependyma. (B) The injury site of the mouse brain after cold injury. Nuclei were stained with DAPI (blue). V, ventricle. Scale bars, 20 μm (A and B).

Figure S6

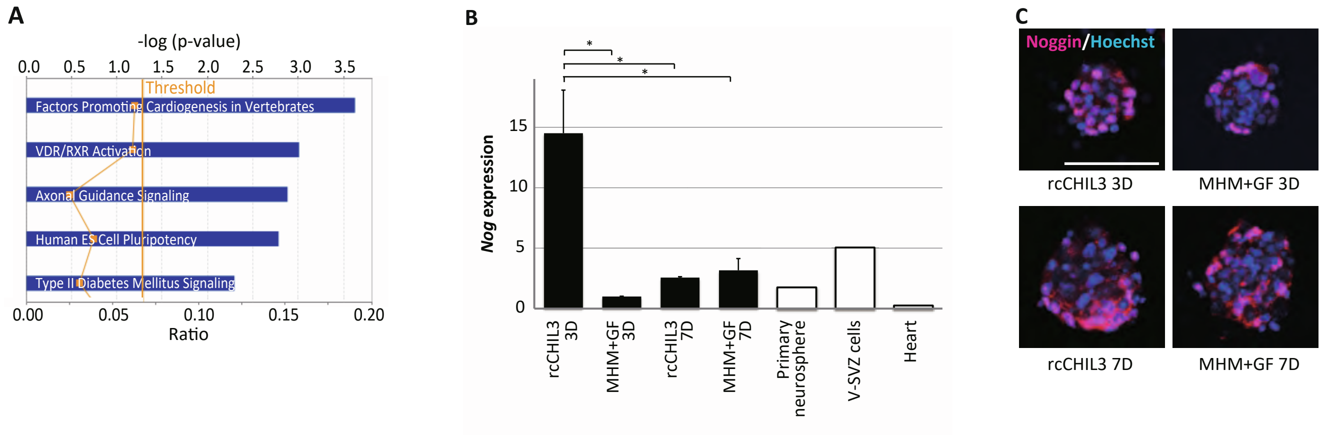

Figure S6. CHIL3-induced canonical pathways and noggin expression in NSCs, related to Figures 6A and 6D. (A) Canonical pathways of the genes classified into Cluster 1. Blue bars, p-value (-Log) calculated with Fisher’ s exact test; orange line, threshold of p = 0.05; orange squares, the ratio of list genes found in each pathway over the total number of genes in that pathway. (B) qRT-PCR of *Nog* gene expression. Expression levels are expressed as relative ratios, with the expression level at MHM+GF 3D set as 1. 2 independent experiments for each. Data are mean ± SEM. *p < 0.05. Samples of primary neurospheres, V-SVZ cells, and heart muscle cells were simultaneously examined as reference data. n = 1 each. (C) Immunocytochemistry of Noggin protein. CHIL3-treated NSCs highly express Noggin protein (red) as compared with MHM+GF-treated neurospheres at 3 DIV. At 7 DIV, Noggin expression illustrates cytoplasmic or intercellular distribution in neurospheres. Nuclei, blue. Scale bar, 20 µm.

Figure S7

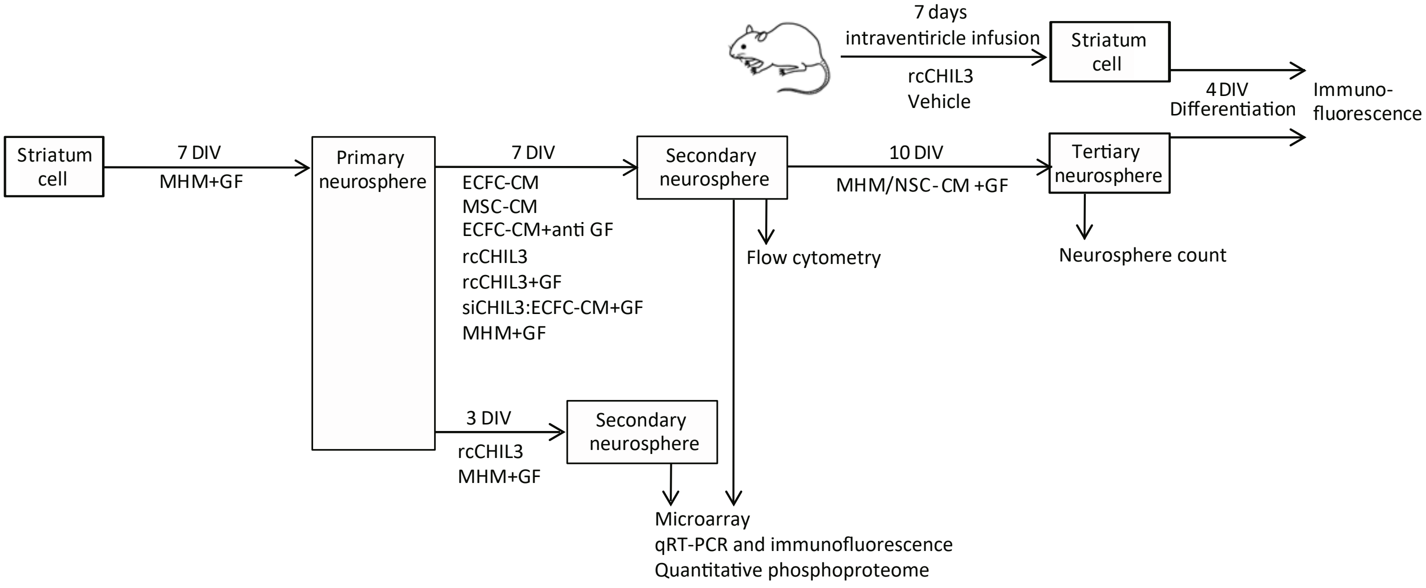

Figure S7. Schematic of cell culture protocol (related to Figures 2A-C, 3A-F, 5, and 6). Since dissected striatum cells are heterogenous, we used primary neurospheres and obtained secondary neurospheres that were treated with distinct media supplemented with test agents. To count neurosphere-initiating cells, which indicate the relative frequency of NSC self-renewal, we dissociated secondary neurospheres, cultured at clonal density with the NSC culture condition, and counted the number of tertiary neurospheres that were generated from the neurosphere-initiating cells in the test media-treated secondary neurosphere. To assess NSC differentiation, since neural stem/progenitor cells migrate away from the niche environment, differentiation culture media were not supplemented with test agents on the assumption as a component of the niche.

Supplementary Dataset 1. Genes and expression values of microarray data and results of cluster analysis (related to Figures 6A, 6D, and 6F). NSCs cultured with rcCHIL3 or control medium (MHM + GF) for 3 days (3D) or 7 days (7D) were processed for microarray analysis. Expression values are expressed as relative signal intensity after global normalization has been performed so that the average signal intensity of all probe sets is equal to 100. Signal detection of gene expression is statistically denoted as present (P), medium (M), or absent (A) by the MAS5 algorithm. Ratios < 1 are expressed as a reciprocal with a minus sign. The reproducibility of the gene expression ratio between two groups was calculated and expressed as ±4 (consistent reproducibility between the two groups when four pairs (2 independent experiments for each group) of the gene expression values are compared) through 0 (no significant difference in gene expression between those two groups).

Supplementary Dataset 2. Phosphorylated proteins, sites, and values of phosphoproteome data and results of cluster analysis (related to Figures 6B, 6C, 6E, and 6G). NSCs cultured with rcCHIL3 or control medium (MHM + GF) for days (3D) or 7 days (7D) were processed for quantitative phosphoproteome analysis. Peak areas of each phosphopeptide were normalized across samples treated with MHM + GF 3D, MHM + GF 7D, rcCHIL3 3D, and rcCHIL3 7D. Ratios are expressed in log2. 2 independent experiments for each group. Analyses were duplicated for each sample.

SUPPLEMENTAL EXPERIMENTAL PROCEDURES

ECFC, mature EC, EC line, MSC, and neurosphere culture

Adherent culture for ECFCs and mature ECs was established on fibronectin-coated 6-well plates (#140675, Nunc, Roskilde, Denmark). Following culture media were used: for ECFCs, endothelial basal medium supplemented with 5% fetal bovine serum (FBS), VEGF, FGF-2, R^3-IGF-1, EGF, hydrocortisone, ascorbic acid, and gentamicin/amphotericin-B (EGM-2-MV Bullet KitCC-3202, Lonza, Walkersville, MD); for committed mature ECs, endothelial maturation medium composed of endothelial basal medium with the supplements listed above but without FGF-2, R^3-IGF-1, and EGF. ECFCs were positive for Ki67, uptake of DiI-Ac-LDL, and rarely positive for von Willebrand factor. both ECFCs and mature ECs were positive for CD31 and VE-cadherin.

Cells of a mouse brain endothelioma cell line were maintained in Dulbecco's Modified Eagle Medium (#12699, Gibco Life Technologies, Carlsbad, CA) supplemented with 10% FBS and 1% penicillin G (10,000 U/mL)-streptomycin sulfate (10,000 mg/mL). The medium was renewed every 3-4 days. At the first passage, 3×10^6 cells were seeded on 6-well plates (9.6 cm^2 per well) with 3 mL of medium and cultured for 4 DIV.

For MSC culture, femurs and tibias of adult mice were dissected free of attached muscles, crushed, and suspended in αMEM (#11900, Gibco) supplemented with 10% FBS and 1% penicillin-streptomycin. Cell suspensions were filtered through a 70-μm filter. Cells (5×10^6 cells/mL) were cultured on non-coated 6-well plates with 3 mL of medium. At 24 h after plating, adherent cells were washed to remove non-adherent cells. The medium was changed weekly, and cells were nearly confluent at 21 DIV.

For neurosphere culture, the striata of embryonal day 14 or adult mouse forebrain were dissected and collected into phosphate-buffered saline (PBS) containing 0.6% glucose. Embryonal striata were mechanically triturated. Adult striata were incubated with trypsin solution for 15 min at 37 °C, triturated, and trypsin inhibitor solution was added. Dissociated cells (2×10^5 cell/mL for embryonic mice, 5000 cells/mL for adult mice) were cultured with serum-free neurosphere culture medium (MHM) supplemented with recombinant human EGF (20 ng/mL) and recombinant human FGF-2 (20 ng/mL) for 7 DIV, and formed floating primary neurospheres. MHM is composed of DMEM-F12 (1:1), glucose (0.6%), glutamine (2 mM), sodium bicarbonate (13.4 mM), HEPES (5 mM), insulin (25 mg/mL), transferrin (100 mg/mL), progesterone (20 nM), sodium selenate (30 nM), and putrescine (60 nM). Neurospheres were mechanically dissociated, clonally (10 cells/μL) cultured with distinct media and formed secondary neurospheres (Figure S7). The following neutralizing antibodies and concentrations were used for the neutralizing experiment (ECFC-CM + anti GF): mouse monoclonal anti-FGF-2 (10 μg/mL; 05-117, Millipore, Billerica, MA) and rabbit polyclonal anti-EGF (20 μg/mL; 06-102, Millipore). For the neurosphere assay, secondary neurospheres were mechanically dissociated into single cells, clonally plated with a 1:1 cocktail of MHM and primary neurosphere culture-conditioned medium supplemented with EGF and FGF-2 (MHM/NSC-CM + GF), and cultured, followed by generation of tertiary neurospheres (Figure S7). The percentage of neurosphere-initiating cells in the population of secondary neurospheres was calculated as 100 multiplied by the number of tertiary neurospheres (diameter > 50 μm) divided by the number of plated cells. Neurosphere counts were performed in a blinded fashion by the same observer.

Tertiary neurospheres or striatum cells from adult mice were allowed to differentiate into the CNS lineage (Figure S7). Neurospheres (one neurosphere/well) or dissociated striatum cells (1×10^4 cells/mL) were plated onto poly-L-ornithine- and fibronectin-coated chamber slides (#5732-008, Iwaki, Chiba, Japan) with MHM plus 1% FBS. Plated neurospheres were then gently triturated several times. Cells were cultured and fixed for immunocytochemistry at 4 DIV. To assess cell proliferation, BrdU (1 μM) was added to the medium.

Immunostaining

A cryostat (FINETEC CM3050S, Leica, Wetzlar, Germany) or a vibratome (VT1200S, Leica, Heidelberg, Germany) were used for coronal sections (14-μm or 50-μm, respectively) of adult

mouse brains. Postnatal days 5 or 16 or adult mouse eyes were isolated for whole-mount samples as previously described (Kubota et al., 2009). Vibratome sections were sequentially preincubated with ice-cold acetone and 1% H_2O_2 in PBS. Whole-mount samples were stored in methanol at -20 °C and rehydrated for staining.

The following primary antibodies and dilutions were used: sheep anti-BrdU (1:100; 20-BS17, Fitzgerald Industries International, Inc., Concord, MA), mouse monoclonal anti-mouse βIII-tubulin (1:1000; T8660, Sigma-Aldrich, St. Louis, MO), mouse monoclonal anti-O4 (1:1000; MAB345, Millipore), mouse monoclonal anti-GFAP (1:100; G3893, Sigma-Aldrich), polyclonal rabbit anti-GFAP (1:2000; Z0334, Dako, Carpinteria, CA), rat monoclonal anti-mouse YM1/Chitinase 3-like 3 (1:50 for immunostaining, 1:500 for immunoblotting, 1:100 for immuno-electron microscopy; MAB2446, R&D Systems, Inc., Minneapolis, MN), goat anti-mouse YM1/Chitinase 3-like 3 (1:1000; AF2446, R&D), rat anti-mouse CD31 (1:10; 550274, BD Biosciences, San Jose, CA), hamster anti-CD31 (1:1000; 2H8, Chemicon, Temecula, CA), goat anti-VE-cadherin (1:10; sc-6458, Santa Cruz Biotechnology, Inc., Santa Cruz, CA), goat anti-mouse Noggin (1:10; AF719, R&D), rabbit anti-Aquaporin 4 (1:200; AB3594, Millipore), mouse monoclonal anti-S-100 β-subunit (1:500; S2532, Sigma-Aldrich), and anti-Doublecortin (1:100; sc-8066, Santa Cruz Biotechnology, Inc.). GFP was not stained with an anti-GFP antibody for sections; crude GFP expression was observed under a fluorescence microscope. Anti-GFP (1:500; Alexa488-conjugated; Molecular Probes, Eugene, OR) was used only for whole-mount samples.

The following signal amplifications were applied for CD31 and VE-cadherin: horseradish peroxidase-conjugated secondary antibody (1:500; Jackson Laboratory, West Grove, PA) and Tyramide Signal Amplification (Renaissance TSA fluorescence system, NEL701-705 (green) or NEL702-705 (red), Perkin Elmer, Waltham, MA). Nuclei were stained with Hoechst 33258 (94403, Sigma-Aldrich) or 4',6-diamidino-2-phenylindole (DAPI, Molecular Probes). Controls lacking the primary antibody were processed in parallel for all histochemistry.

After staining, cells and sections were observed with a fluorescence microscope (BX-61, Olympus, Tokyo, Japan; or AxiCom, Zeiss Deutschland, Oberkochen, Germany) or a confocal laser microscope (LSM510 META, Zeiss; LSM5 Pascal, Zeiss; or FV1000, Olympus). Florescence images were prepared from data files with AxioVision 4 software or Zeiss Image Browser Ver. 3.5 software, and with Adobe Photoshop Ver. 12.1 software (San Jose, CA). Linear adjustments to brightness or contrast were applied to the entire image when necessary. The numbers of βIII-tubulin-, O4-, GFAP-, and BrdU-positive cells were counted in five non-overlapping visual fields at a magnification of 400×. Cell counts were performed in a blinded fashion by the same observer. The number of BrdU- and Doublecortin-positive cells in the right V-SVZ (the side of the intraventricle infusion) of eight serial in every tenth section was counted and summed in a blinded fashion by the same observer. BrdU-positive cells in the granule cell layer of the olfactory bulb (both injected side and contralateral side) were counted with the Dynamic Cell Count software under a fluorescence microscope (BZ-9000, Keyence, Osaka, Japan). A region of interest was set manually for the granule cell layer. Counts of three in every tenth section were summed.

Flow cytometry

Single-cell suspensions of secondary neurospheres were processed for flow cytometry. Cells were stained with fluorescein isothiocyanate anti-human CD15 (555401, BD Biosciences) or phycoerythrin anti-human CD15 (55402, BD Biosciences). PI (2 μg/mL) was added to discriminate dead cells from live cells. Cell suspensions were analyzed with a FACS Vantage (Becton Dickinson). Cells obtained from E/nestin:dVenus transgenic mouse embryos were assayed for crude Venus expression. The proportions of CD15- and Venus-positive cells among all live cells were obtained by analyzing the cumulative data from each animal.

ELISA

The following ELISA kits were used according to the manufacturer's instructions: Quantikine Human FGF Basic (DFB50), Quantikine Mouse EGF (MEG00), Quantikine Mouse VEGF (MMV00), Quantikine Human BDNF (DBD00), and Quantikine Mouse Chitinase 3-like 3/ECF-L (MC3L30, all kits from R&D).

For CSF collection, mice were anesthetized with an inhalant anesthetic and placed on a stereotactic frame (SR-5, Narishige, Tokyo, Japan) in a position that flexed the neck. A midline incision on the neck was made, and then the skull of the posterior part and the upper cervical vertebrae were exposed. CSF was gently drawn up at the puncture at the dura mater of the cisterna magna. CSF obtained from one animal or a pooled sample from a few animals was processed for ELISA since the volume of CSF obtained was less than 50 µL per animal.

Supernates of the blood were collected as serum after coagulation and centrifugation. Brain cells were rinsed with PBS, chopped into 1-2 mm pieces, and cultured in Roswell Park Memorial Institute medium with 2 mM L-glutamine (11875-093, Gibco) supplemented with 10% FBS and penicillin-streptomycin. Culture supernates of brain cells were removed at 4 DIV and assayed.

Immuno-electron microscopy

Frozen sections were incubated with rat monoclonal anti-mouse CHIL3 primary antibody followed by incubation with biotin-conjugated donkey anti-rat secondary antibody (1:1000; 712-065, Jackson ImmunoResearch Laboratories, Inc., West Grove, PA) and with Alexa488- and nanogold-conjugated streptavidin (1:100; A-24926, Molecular Probes Invitrogen, Carlsbad, CA). After enhancement with Silver Enhancement Kit (HQ Silver, Catalog number 2012, Nanoprobes Inc., Yaphank, NY), sections were post-fixed, dehydrated, and embedded in Epon. Ultrathin sections were stained with uranyl acetate and lead citrate and observed under a transmission electron microscope (model 1230, JEOL, Tokyo, Japan). Images were taken with Digital Micrograph 3.3 (Gatan Inc., Pleasanton, CA).

Differential proteomics

ECFC-CM and MSC-CM were pretreated with Multiple Affinity Removal Spin Cartridges (Mouse 3, Agilent Technologies, Santa Clara, CA) according to the manufacturer's instructions to remove albumin, IgG, and transferrin. Proteins were extracted and processed. A pooled sample (1:1 weight mixture of ECFC-CM and MSC-CM), ECFC-CM, and MSC-CM were labeled with Cy2, Cy3, and Cy5 (GE Healthcare Biosciences, Uppsala, Sweden), respectively. Protein aliquots (200 µg) were first separated according to isoelectronic point with Multiphore II (GE Healthcare Biosciences) and Immobilized pH Gradient Strips (24 cm, pI3-10, GE Healthcare Biosciences). Sodium dodecyl sulfate polyacrylamide gel electrophoresis was applied to the second dimension, separating the proteins by mass, with an Ettan DALT II Electrophoresis System (GE Healthcare Biosciences). The gel images were acquired with Typhoon (GE Healthcare Biosciences) and processed for spot detection, background subtraction, normalization, quantitation of spots in images from a single gel, spot matching on multiple gels, and statistical analysis of changes in protein abundance with DeCyder 2D Differential Analysis Software (GE Healthcare Biosciences).

A 600-µg protein extract from ECFC-CM was loaded for two-dimensional electrophoresis. Spots on the gel were stained with Sypro Ruby Protein Gel Stain (#S12001, Molecular Probes Invitrogen). The gel was imaged with MasterImager (GE Healthcare Biosciences). Spots of interest were matched by DeCyder 2D Differential Analysis Software (GE Healthcare Biosciences), picked from the gel with an Ettan Spot Picker (GE Healthcare Biosciences), digested, and analyzed via nanoLC-MS/MS with the CapLC system (Waters, Milford, MA) and the Micromass Q-Tof Micro Mass Spectrometer (Waters) with MassLynx Software (Waters).

rcCHIL3

Plasmid DNA containing the sequence encoding *Chil3* was obtained from *Escherichia coli* DH10B TonA with the pDNR-LIB vector encoding the mouse *Chil3* cDNA (IRALp962M1053Q, Source

BioScience imaGenes, Berlin, Germany) with a QIAprep Spin Miniprep Kit (27104, QIAGEN Inc., Valencia, CA) and transferred into the pLP-CMV-Myc acceptor vector (631603, Clontech Laboratories, Inc., Mountain View, CA). DH5α cells were transfected with the acceptor vector and transformed by heat shock. Plasmid DNA was retrieved from the DH5α competent cells with a QIAprep Spin Miniprep Kit (27104, QIAGEN) and scaled up with the Qiaprep Spin Maxiprep Kit (10262, QIAGEN). Expression vector pCS2+ harboring the gene encoding Venus was used for mock experiments. HEK293T cells were cultured on poly-L-ornithine-coated 10-cm plastic dishes for 1 DIV. Then, a mixture of *Chil3* DNA (6 µg), Opti-MEM I Reduced-Serum Medium (800 µL, 31985-070, Gibco), and GeneJuice Transfection Reagent (15 µL, 70967, Novagen, Darmstadt, Germany) was added to 15 mL of culture medium in a 10-cm dish. The cells were cultured for another 1 DIV. The culture medium was then changed to MHM 1 day before obtaining rcCHIL3.

CHIL3 knock-down

ECFCs were established in a 6-well plate as described above. At 21 DIV, a siRNA complex of *Chil3* consisting of siRNA (4 µg/well), Opti-MEM I Reduced-Serum Medium (80 µL/well, 31985-070, Gibco), and X-tremeGENE siRNA Transfection Reagent (20 µL/well, 04 476 093 001, Roche Diagnostics, Mannheim, Germany) was added to the culture medium, and the cells were cultured for another 1 DIV. The culture medium was changed and the conditioned medium, siCHIL3: ECFC-CM, was then obtained. siRNA against the gene encoding glyceraldehyde 3-phosphate dehydrogenase (GAPDH; Silencer FAM, AM4650, Ambion Invitrogen) and negative control siRNA provided by the Sigma Genosys siRNA Service were used as controls.

Microarray analysis

Total RNA was isolated from rcCHIL3-treated or MHM + GF-treated secondary neurospheres with the Qiagen RNeasy Mini Kit (74104, QIAGEN, Hilden, Germany). DNA microarray analysis using Affymetrix Gene Chip technology was performed as described previously (Heishi et al., 2008; Heishi et al., 2006; Ishida et al., 2002; Matsui et al., 2012). Briefly, 100 ng of total RNA was used as a template for cDNA synthesis, and biotin-labeled cRNA was synthesized with the 3' IVT Express Kit (901228, Affymetrix, Santa Clara, CA). After generating the hybridization cocktails, hybridization to the DNA microarray (900496, GeneChip Mouse Genome 430 2.0 Array, Affymetrix) (Lockhart et al., 1996) and fluorescent labeling were performed. The microarrays were then scanned with a GeneChip Scanner 3000 7G System (Affymetrix). Data analysis was carried out using GeneChip Operating Software 1.04 (Affymetrix). Signal detection and quantification were performed using the MAS5 algorithm with default settings.

For the clustering analysis, signals were normalized, calculated, and visualized with Spotfire DecisionSite System 9.1.2 (TIBCO, Palo Alto, CA). Principal component analysis was carried out with Spotfire DecisionSite 9.1.2 using normalized data. Pathway analysis was performed with Ingenuity Pathway Analysis ver.14197757 (Ingenuity Systems, Redwood City, CA).

qRT-PCR

qRT-PCR for *Nog* was performed according to the manufacturer’s instructions (QIAGEN). Total RNA was isolated from rcCHIL3-treated or MHM + GF-treated secondary neurospheres, primary neurospheres, V-SVZ cells, and heart muscle cells (from embryonal day 14 mouse embryos) with the miRNeasy Mini Kit (217004, QIAGEN), and was treated with an RNase-free DNase set (79254, Qiagen) to remove contaminating genomic DNA. We used the QuantiFast SYBR Green PCR Kit (204052, Qiagen) and the ABI StepONePlus instrument and software v2.1 (Applied Biosystems Life Technologies, Carlsbad, CA) for qRT-PCR with the following qPCR program: 95 °C for 5 min and 40 cycles of 95 °C for 10 s, 60 °C for 30 s, and 72 °C for 15 s. Melting-curve analysis was routinely used for each reaction. The gene encoding GAPDH was run in parallel as an internal control for each reaction set.

Quantitative phosphoproteome analysis

Proteins were extracted from 100,000 cells using 12 mM sodium deoxycholate and 12 mM sodium lauroyl sarcosinate, and digested with Lys-C and trypsin (Masuda et al., 2009). Phosphopeptides were enriched by aliphatic hydroxy acid-modified metal oxide chromatography with titania (Sugiyama et al., 2007) and analyzed by nanoLC-MS/MS using an LTQ-Orbitrap instrument (Thermo Fisher Scientific, Bremen, Germany). Peptides and proteins were identified using Mascot version 2.3 (Matrix Science, London, UK) with the Swiss-Prot database. Label-free quantitation was performed based on the peak areas of extracted ion chromatograms for identified phosphopeptides using Mass Navigator (Mitsui Knowledge Industry, Tokyo, Japan). Two independent experiments for each group. Analyses were duplicated for each sample.

For the clustering analysis, the peak area of each phosphopeptide was normalized across samples of the culture groups. The normalized peak areas and the peak area ratios were subjected to cluster analysis with Cluster version 2.11 (http://rana.lbl.gov/EisenSoftware.htm) (Eisen et al., 1998).

***In vivo* intraventricle infusion**

Adult mice were anesthetized with an inhalant anesthetic and placed on a stereotactic frame (SR-5, Narishige). A cannula (0008851 ALZET Brain Infusion Kit 3, DURECT Co.) was implanted stereotaxically at the coordinates of anteroposterior = 0 mm, mediolateral = –1.2 mm, and dorsoventral = –2.3 mm relative to the bregma, and glued with a cyanoacrylate adhesive onto the skull. A mini-osmotic pump (0.5 µL/h, model 1007D ALZET micro-osmotic pump, DURECT Co., Cupertino, CA) was attached to the cannula and implanted subcutaneously into the dorsal flank. After surgery, mice were housed before sacrifice.

For the immunohistochemical preparations, mice were transcardially perfused with PBS, followed by 4% paraformaldehyde. Forebrains were removed and processed. For *ex vivo* culture, forebrains were aseptically removed and processed.

REFERENCES

Eisen, M.B., Spellman, P.T., Brown, P.O., and Botstein, D. (1998). Cluster analysis and display of genome-wide expression patterns. Proc Natl Acad Sci U S A *95*, 14863-14868.

Heishi, M., Hayashi, K., Ichihara, J., Ishikawa, H., Kawamura, T., Kanaoka, M., Taiji, M., and Kimura, T. (2008). Comparison of gene expression changes induced by biguanides in db/db mice liver. The Journal of toxicological sciences *33*, 339-347.

Heishi, M., Ichihara, J., Teramoto, R., Itakura, Y., Hayashi, K., Ishikawa, H., Gomi, H., Sakai, J., Kanaoka, M., Taiji, M.*, et al.* (2006). Global gene expression analysis in liver of obese diabetic db/db mice treated with metformin. Diabetologia *49*, 1647-1655.

Ishida, N., Hayashi, K., Hoshijima, M., Ogawa, T., Koga, S., Miyatake, Y., Kumegawa, M., Kimura, T., and Takeya, T. (2002). Large scale gene expression analysis of osteoclastogenesis in vitro and elucidation of NFAT2 as a key regulator. J Biol Chem *277*, 41147-41156.

Kubota, Y., Takubo, K., Shimizu, T., Ohno, H., Kishi, K., Shibuya, M., Saya, H., and Suda, T. (2009). M-CSF inhibition selectively targets pathological angiogenesis and lymphangiogenesis. The Journal of experimental medicine *206*, 1089-1102.

Lockhart, D.J., Dong, H., Byrne, M.C., Follettie, M.T., Gallo, M.V., Chee, M.S., Mittmann, M., Wang, C., Kobayashi, M., Horton, H.*, et al.* (1996). Expression monitoring by hybridization to high-density oligonucleotide arrays. Nature biotechnology *14*, 1675-1680.

Masuda, T., Saito, N., Tomita, M., and Ishihama, Y. (2009). Unbiased quantitation of Escherichia coli membrane proteome using phase transfer surfactants. Mol Cell Proteomics *8*, 2770-2777.

Matsui, T., Takano, M., Yoshida, K., Ono, S., Fujisaki, C., Matsuzaki, Y., Toyama, Y., Nakamura, M., Okano, H., and Akamatsu, W. (2012). Neural stem cells directly differentiated from partially reprogrammed fibroblasts rapidly acquire gliogenic competency. Stem Cells *30*, 1109-1119.

Sugiyama, N., Masuda, T., Shinoda, K., Nakamura, A., Tomita, M., and Ishihama, Y. (2007). Phosphopeptide enrichment by aliphatic hydroxy acid-modified metal oxide chromatography for nano-LC-MS/MS in proteomics applications. Mol Cell Proteomics *6*, 1103-1109.
