# Supplementary material for: Chitinase-like protein 3: A novel niche factor for mouse neural stem cells
Source: Stem Cell Reports. 2022 Nov 10;17(12):2704–17. doi: 10.1016/j.stemcr.2022.10.012 (PMC9768575; doi:10.1016/j.stemcr.2022.10.012)
Supplement: Document S2. Article plus supplemental information [file mmc4.pdf]

Stem Cell Reports

Article

OPEN ACCESS

Chitinase-like protein 3: A novel niche factor for mouse neural stem cells

Jun Namiki,[1,2,8,*] Sayuri Suzuki,[1,2] Shinsuke Shibata,[2,9] Yoshiaki Kubota,[3] Naoko Kaneko,[4,10] Kenji Yoshida,[2,5] Ryo Yamaguchi,[2,5] Yumi Matsuzaki,[2,11] Takeshi Masuda,[6,12] Yasushi Ishihama,[6,7] Kazunobu Sawamoto,[4] and Hideyuki Okano[2,*]

[1]Department of Emergency and Critical Care Medicine, Keio University School of Medicine, Shinjuku, Tokyo 160-8582, Japan
[2]Department of Physiology, Keio University School of Medicine, Shinjuku, Tokyo 160-8582, Japan
[3]Department of Anatomy, Keio University School of Medicine, Shinjuku, Tokyo 160-8582, Japan
[4]Department of Developmental and Regenerative Neurobiology, Institute of Brain Science, Nagoya City University Graduate School of Medical Sciences, Nagoya, Aichi 467-8601, Japan
[5]Sumitomo Pharma Co. Ltd., Osaka, Osaka 541-0045, Japan
[6]Institute for Advanced Biosciences, Keio University, Tsuruoka, Yamagata 997-0017, Japan
[7]Graduate School of Pharmaceutical Sciences, Kyoto University, Kyoto 606-8501, Japan
[8]Present address: Department of Emergency Medicine, KKR Tachikawa Hospital, Tachikawa, Tokyo 190-8531, Japan
[9]Present address: Division of Microscopic Anatomy, Graduate School of Medical and Dental Sciences, Niigata University, Niigata 951-8510, Japan
[10]Present address: Laboratory of Neuronal Regeneration, Graduate School of Brain Science, Doshisha University, Kyotanabe-shi, Kyoto 610-0394, Japan
[11]Present address: Department of Life Science, Shimane University Faculty of Medicine, Izumo, Shimane 693-8501, Japan
[12]Present address: Department of Pharmaceutical Microbiology, Faculty of Life Sciences, Kumamoto University, Chuo-ku, Kumamoto 862-0973, Japan
*Correspondence: namiki@med.keio.ac.jp (J.N.), hidokano@a2.keio.jp (H.O.)
https://doi.org/10.1016/j.stemcr.2022.10.012

SUMMARY

The concept of a perivascular niche has been proposed for neural stem cells (NSCs). This study examined endothelial colony-forming cell (ECFC)-secreted proteins as potential niche factors for NSCs. Intraventricle infusion with ECFC-secreted proteins increased the number of NSCs. ECFC-secreted proteins were more effective in promoting NSC self-renewal than marrow stromal cell (MSC)-secreted proteins. Differential proteomics analysis of MSC-secreted and ECFC-secreted proteins was performed, which revealed chitinase-like protein 3 (CHIL3; also called ECF-L or Ym1) as a candidate niche factor for NSCs. Experiments with recombinant CHIL3, small interfering RNA, and neutralizing antibodies demonstrated that CHIL3 stimulated NSC self-renewal with neurogenic propensity. CHIL3 was endogenously expressed in the neurogenic niche of the brain and retina as well as in the injured brain and retina. Transcriptome and phosphoproteome analyses revealed that CHIL3 activated various genes and proteins associated with NSC maintenance or neurogenesis. Thus, CHIL3 is a novel niche factor for NSCs.

INTRODUCTION

During embryonic development, neural stem cells (NSCs) in the ventricular zone of the mammalian brain undergo symmetric division (self-renewal) to expand the NSC pool. Next, the expanded NSCs undergo asymmetric division to generate neurons that migrate to the parenchyma. NSC expansion and neurogenesis are closely associated with vascular development in the brain. Neurogenesis from early NSCs and proliferation and differentiation of angioblasts into endothelial cells (ECs) are completed by midgestation. Late NSCs with neurogenic propensity exhibit decreased self-renewal capacity and undergo differentiation into glia, except in two neurogenic niches, throughout adult life: the ventricular-subventricular zone (V-SVZ) of the lateral ventricle and the subgranular zone (SGZ) of the hippocampal dentate gyrus. Adult NSCs in the V-SVZ undergo self-renewal and divide into transit-amplifying cells, which subsequently generate neuroblasts. The neuroblasts migrate from the V-SVZ into the olfactory bulb and differentiate into interneurons (reviewed in Akter et al., 2021). The correlation between self-renewal of early NSCs with neurogenic propensity and vasculogenesis of mesoderm-derived angioblasts during development indicates that mesodermal bone marrow-derived endothelial colony-forming cells (ECFCs; reviewed in Critser and Yoder, 2010) secrete microenvironment-related molecules that maintain the NSC niche.

The concept of a perivascular niche has been proposed for the adult mammalian SGZ (Palmer et al., 2000) and V-SVZ (Shen et al., 2008; Tavazoie et al., 2008). The local microvascular bed is important for providing a permissive environment for NSC expansion and neurogenesis. For example, the long basal processes of NSCs directly contact blood vessels, which suggests that NSCs receive signals from the vessels (reviewed in Obernier and Alvarez-Buylla, 2019). Various ECs that secrete soluble factors have been reported to modulate NSC activity and regulate NSC self-renewal.

This study demonstrated that the ECFC-secreted protein chitinase-like protein 3 (CHIL3) is a critical factor for the NSC niche. Experiments with the recombinant protein, small interfering RNA (siRNA), and neutralizing antibodies revealed that CHIL3 promoted the self-renewal of NSCs with neurogenic propensity. This study demonstrated endogenous expression of CHIL3 in the brain and retina.

This is an open access article under the CC BY license (http://creativecommons.org/licenses/by/4.0/).

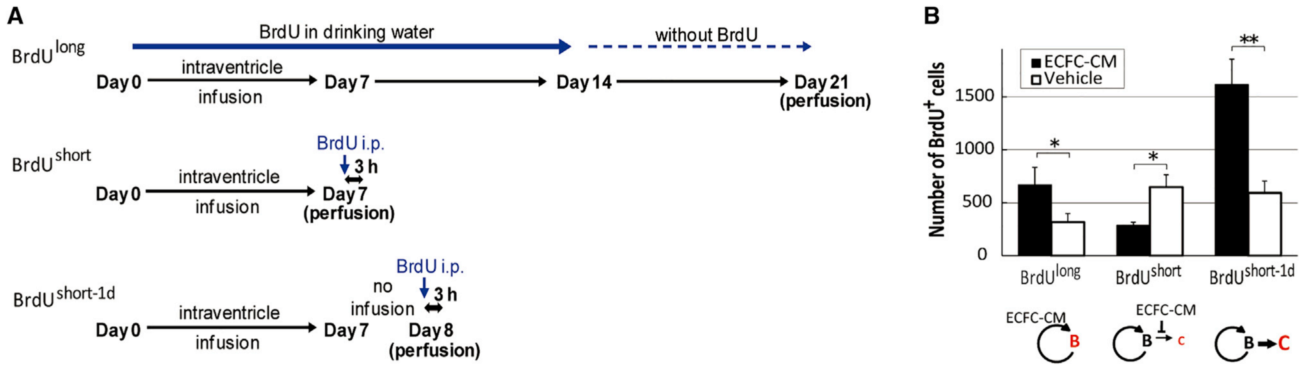

Figure 1. Endothelial colony-forming cell (ECFC)-secreted factors increase the number of neural stem cells (NSCs) in the adult ventricular-subventricular zone (V-SVZ)

(A) Intraventricular infusion and distinct regimen of BrdU administration.

(B) Numbers of BrdU-positive cells on the infusion side of the V-SVZ after infusion with CM of ECFCs (ECFC-CM) or vehicle. Red letters indicate BrdU-labeled cell types based on long-term ($BrdU^{long}$) or short-term ($BrdU^{short}$ and $BrdU^{short-1d}$) labeling. B, NSCs; C, transit-amplifying cells.

See also Figure S1 for immunohistochemistry images. 3–7 independent experiments for each group; $*p < 0.05$, $**p < 0.01$.

CHIL3 activated various genes and proteins associated with NSC maintenance or neurogenesis in NSCs.

RESULTS

ECFC-secreted factors promote NSC self-renewal

ECFC-secreted soluble factors that promote NSC expansion *in vivo* were examined. The conditioned medium (CM) obtained from ECFCs (ECFC-CM) were infused into the adult mouse lateral ventricle for 7 days, followed by administration of bromodeoxyuridine (BrdU) to label NSCs (long labeling [$BrdU^{long}$]; Figure 1A) that retain BrdU for a prolonged duration (Doetsch et al., 1999) or transit-amplifying cells that retain BrdU for a short duration (short labeling [$BrdU^{short}$ and $BrdU^{short-1d}$]; Figure 1A). The counts of NSCs in the V-SVZ of the ECFC-CM-treated group were significantly higher than those in the V-SVZ of the vehicle-treated group ($p < 0.05$; Figures 1B and S1). This indicated that ECFC-secreted factors promote NSC expansion. A single injection of BrdU labeled the proliferating transit-amplifying cells. In contrast to NSCs, the counts of transit-amplifying cells in the ECFC-CM-treated group were lower than those in the vehicle-treated control group ($p < 0.05$, $BrdU^{short}$). On day 1 after ECFC-CM infusion cessation, the number of transit-amplifying cells markedly increased, which indicated that large numbers of transit-amplifying cells were derived from accumulated NSCs ($p < 0.01$, $BrdU^{short-1d}$). Thus, infusion of ECFC-CM *in vivo* increased the number of NSCs and suppressed generation of transit-amplifying cells until cessation of the infusion. These findings indicate that ECFC-CM promotes NSC self-renewal in the V-SVZ.

Differential proteomics analysis identified CHIL3 as a novel ECFC-specific niche factor for NSCs

Next, the principal component of ECFC-CM involved in NSC self-renewal was examined. Self-renewal and multipotency are definitive characteristics of NSCs. The multipotency of neurospheres cultured with ECFC-CM was confirmed (Figure 2A). An *in vitro* neurosphere assay was performed to assess the self-renewal capacity of NSCs, and the results are expressed as the number of neurospheres (tertiary neurospheres; see culture protocol in Figure S7) generated from neurosphere-initiating cells in ECFC-CM-treated neurospheres (secondary neurospheres) (Soares et al., 2021). The percentage of neurosphere-initiating cells in the group treated with ECFC-CM supplemented with neutralizing anti-fibroblast growth factor 2 (FGF-2) and anti-epidermal growth factor (EGF) antibodies (ECFC-CM + anti-growth factor [GF]) was significantly higher than that in the group treated with a control medium containing medium hormone mix (MHM) supplemented with FGF-2 and EGF (GF), an appropriate culture medium for NSC expansion ($p < 0.05$; Figure 2B). This indicated that ECFC-CM comprised a distinct factor that was more effective in increasing the number of neurosphere-initiating cells than this combination and concentration of these GFs. The percentage of neurosphere-initiating cells in the ECFC-CM-treated group was higher than that in the ECFC-CM + anti-GF-treated group ($p < 0.05$; Figure 2B). Thus, the NSC self-renewal-promoting effect of ECFC-CM was not dependent on GF. GF potentiated the NSC self-renewal-promoting effects of ECFC-CM. The results of an enzyme-linked immunosorbent assay revealed the presence of GF in ECFC-CM at picogram concentrations (FGF-2 3.4 and EGF 20.6 pg/mL on average; Figure S2).

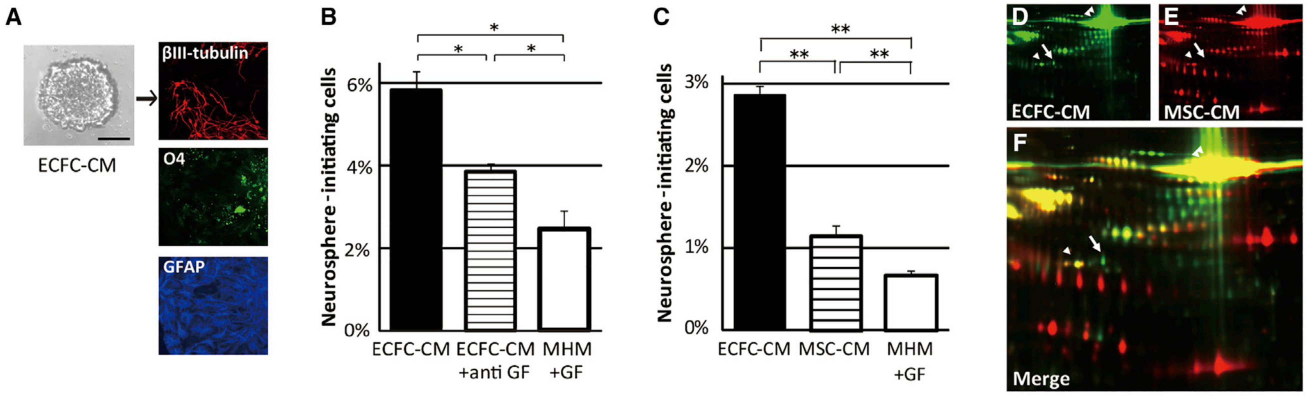

G

Rank	Master No.	ECFC-CM/ MSC-CM ratio	t-test	Protein Name
1	1302	22.3	0.0005	Chitinase-like protein 3
2	822	20.37	0.0022	Serotransferrin
3	840	11.91	0.00091	Serotransferrin
4	84	9.56	0.0068	Serotransferrin
5	252	9.13	0.0071	Serotransferrin
6	821	8.69	0.001	Serotransferrin
7	988	7.05	0.0021	No HIT
8	117	6.66	0.0025	Serotransferrin
9	1219	6.55	0.0051	Pigment epithelium-derived factor
10	960	6.47	0.0013	No HIT
11	1231	6.35	0.005	N.E.
12	992	5.96	0.0027	No HIT
13	538	5.87	0.0017	Gelsolin
14	546	5.23	0.0026	Gelsolin

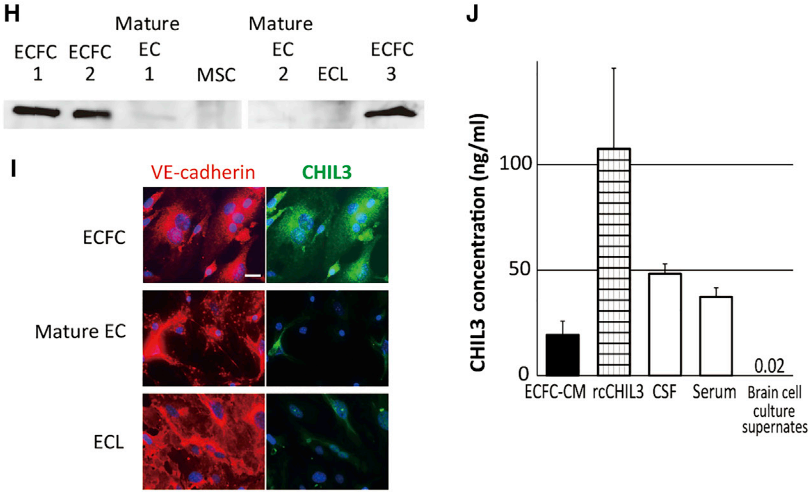

Figure 2. ECFC-secreted factors promote NSC self-renewal more effectively than marrow stromal cell (MSC)-secreted factors *in vitro*. Differential proteomics analysis of these factors

(A) ECFC-CM-treated neurospheres exhibiting multipotency and differentiating into the following three neural lineages: neurons (βIII-tubulin, red), oligodendrocytes (O4, green), and astrocytes (GFAP, blue). See also Figure S7 for the culture protocol. Scale bars, 50 μm.

(B and C) Percentages of neurosphere-initiating cells in neurospheres cultured with ECFC-CM, growth factor (GF)-depleted ECFC-CM (ECFC-CM + anti-GF), or control medium with GF (MHM + GF) (B) as well as with ECFC-CM, MSC-CM, or MHM + GF (C). 3 (B) and 20 (C) independent experiments; $^{*}p < 0.05$, $^{**}p < 0.01$.

(D–F) 2D-DIGE analysis of spots from ECFC-CM (green in D and F) and MSC-CM (red in E and F). See also grayscale images in Figure S3. Arrow, the most ECFC-CM-specific spot (spot 1,302 in G); arrowheads and double arrowheads, nonspecific spots of β-actin and transferrin, respectively.

(G) Proteins identified using MS. The top 14 ECFC-CM-specific spots for which the protein abundance ratio (ECFC-CM/MSC-CM) was greater than 5 ($p < 0.01$) were processed. Serotransferrin is derived from the serum in the medium. Red letters indicate ECFC-CM-specific proteins. No HIT, no database match; N.E., not examined because of technical difficulties.

(H–J) CHIL3 expression analysis using immunoblotting (H), immunostaining (I), and enzyme-linked immunosorbent assay (J). ECL, endothelial cell line. 2–5 independent experiments for each group (J). Scale bars, 20 μm.

The results of the neurosphere assay demonstrated that the ability of ECFC-CM to increase the percentage of neurosphere-initiating cells was higher than that of marrow stromal cell (MSC)-CM ($p < 0.01$; Figure 2C). Hence, ECFC-CM and MSC-CM (control) were subjected to differential proteomics analyses to identify the candidate ECFC-specific niche factors for NSCs. MSCs, also called mesenchymal stem cells, are derived from the bone marrow and secrete various GFs, including EGF and VEGF. EGF and VEGF were detected in MSC-CM and ECFC-CM (Figure S2). Two-dimensional fluorescence difference gel electrophoresis (2D-DIGE) analysis revealed that the expression levels of approximately 2,000 proteins with distinct molecular weights and electric charges were different between

ECFC-CM and MSC-CM (Figures 2D–2F and S3A–S3C). Fourteen ECFC-CM-specific spots exhibiting the highest protein abundance ratio were analyzed, and their peptide composition was identified using nanoscale liquid chromatography and tandem mass spectrometry (nanoLC-MS/MS) (Figures 2G and S3D). The most ECFC-CM-specific protein was CHIL3, also called ECF-L or Ym1 (Figures 2G, S3E, and S3F). In total, 14 spots were examined and identified Pigment epithelium-derived factor (PEDF) (Castro-Garcia et al., 2015; Ramirez-Castillejo et al., 2006) and Gelsolin (Kronenberg et al., 2010). Immunoblotting and immunocytochemical analyses confirmed that CHIL3 expression was high in the culture supernatant or the cytoplasm of ECFCs but faint or absent in the culture supernatant or the cytoplasm of mature ECs, EC lines, and MSCs (Figures 2H and 2I). Endogenous CHIL3 was detected in the cerebrospinal fluid (CSF) and serum (Figure 2J).

CHIL3 stimulates NSC self-renewal and neurogenesis

To determine the function of CHIL3 in NSCs, recombinant CHIL3 (rcCHIL3) was used. CM was prepared from ECFCs transfected with *Chil3* siRNA (siChil3:ECFC-CM) for a *Chil3* knockdown experiment (Figure S4A). The results of the neurosphere assay revealed that the percentage of neurosphere-initiating cells in the group treated with culture medium containing rcCHIL3 was similar to that in the group treated with ECFC-CM but higher than that in the group treated with MHM + GF ($p < 0.01$, Figure 3A; $p < 0.05$, Figure S4B). Culture medium supplemented with rcCHIL3 generated neurospheres even in the absence of GF. The percentage of neurosphere-initiating cells in the siChil3:ECFC-CM + GF-treated group was similar to that in the MHM + GF-treated group (Figure 3B). This indicated that CHIL3 is a principal component of ECFC-CM that promotes NSC self-renewal. Expression of the NSC marker in rcCHIL3-treated neurospheres was confirmed using flow cytometry analysis. E/nestin:dVenus transgenic mouse embryos were used, in which *Nes* expression is upregulated only in neural stem/progenitor cells during G1-S phase (Sunabori et al., 2008). The percentages of CD15-positive and Venus-positive cells in rcCHIL3-treated neurospheres were higher than those in the MHM + GF-treated control ($p < 0.01$, Figure 3C; $p < 0.05$, Figure S4C). Next, the effect of rcCHIL3 on neurogenesis was examined. rcCHIL3-treated NSCs were dissociated, cultured, and allowed to differentiate in the absence of rcCHIL3. The number of cells exhibiting the neuronal phenotype in the rcCHIL3-treated group was higher than that in the MHM + GF-treated control group ($p < 0.01$, Figures 3D and S4D; for the culture protocol, see Figure S7). Neurospheres treated with ECFC-CM also exhibited enhanced neurogenesis ($p < 0.05$). However, the number of cells exhibiting the neuronal phenotype in the siChil3:ECFC-CM + GF-treated group was similar to that in the MHM + GF-treated control group.

The results of *in vitro* experiments indicated that NSCs cultured in the presence of rcCHIL3 promoted neurogenesis. Next, an *in vivo* infusion experiment was performed. After rcCHIL3 infusion for 7 days, V-SVZ cells were dissected and allowed to differentiate with BrdU *ex vivo*. The number of BrdU-incorporating cells in the rcCHIL3-infused group was higher than that in the vehicle-infused control group ($p < 0.05$; Figure 3E). An increased number of dividing V-SVZ cells exhibited the neuronal phenotype rather than the glial phenotype ($p < 0.05$; Figures 3F and S4E). The *in vivo* effects of CHIL3 on NSC self-renewal and neurogenesis were examined using the same protocol as the ECFC-CM infusion experiment shown in Figure 1A. The number of V-SVZ NSCs labeled with $BrdU^{long}$ increased after infusion of rcCHIL3 ($p < 0.05$) but decreased after infusion of anti-CHIL3 neutralizing antibodies ($p < 0.01$; Figure 3G). Similar to ECFC-CM infusion (Figure 1B), production of proliferative transit-amplifying cells that were labeled with a single BrdU administration ($BrdU^{short}$) was suppressed at the end of rcCHIL3 infusion ($p < 0.05$) but increased 1 day after terminating rcCHIL3 infusion ($BrdU^{short\text{-}1d}$) ($p < 0.01$; Figure 3G). The number of DCX/BrdU double-positive cells in the rcCHIL3-infused group was higher than that in the vehicle control-infused group on day 3 after termination of the rcCHIL3 infusion ($BrdU^{short\text{-}3d}$) ($p < 0.05$; Figure 3G). This indicated that the increased number of transit-amplifying cells resulted in an enhanced number of neuroblasts in the V-SVZ. Neuroblasts retain the $BrdU^{long}$ phenotype as they differentiate, migrate, and cease dividing. The number of BrdU-positive cells of the olfactory bulb, which were considered to be progenies of neuroblasts that migrated from the V-SVZ, on the infused side of the rcCHIL3-infused group was higher than that of the vehicle control group ($p < 0.05$; Figure 3H). These *in vivo* infusion experiments indicate that rcCHIL3 promoted NSC self-renewal and consequently increased the number of NSCs in the V-SVZ. Subsequently, neuroblasts were generated from the NSCs and migrated into the olfactory bulb.

CHIL3 is endogenously expressed in the neurogenic niches of the brain and retina

Immunohistochemistry analysis demonstrated the endogenous distribution of CHIL3 in the close vicinity of the SVZ vasculature (exclusively on the lateral side of the ventricle, where adult NSCs reside) (Figures 4D–4F) as well as in the vasculature of the SGZ, another neurogenic niche in the brain (Figures 4A and 4C), but not in the cortex (Figure 4B). VEFGR2 has been reported to be expressed in ECFCs (Chambers et al., 2021). Hence, the expression patterns of VEGF receptors were examined. The V-SVZ harbored a

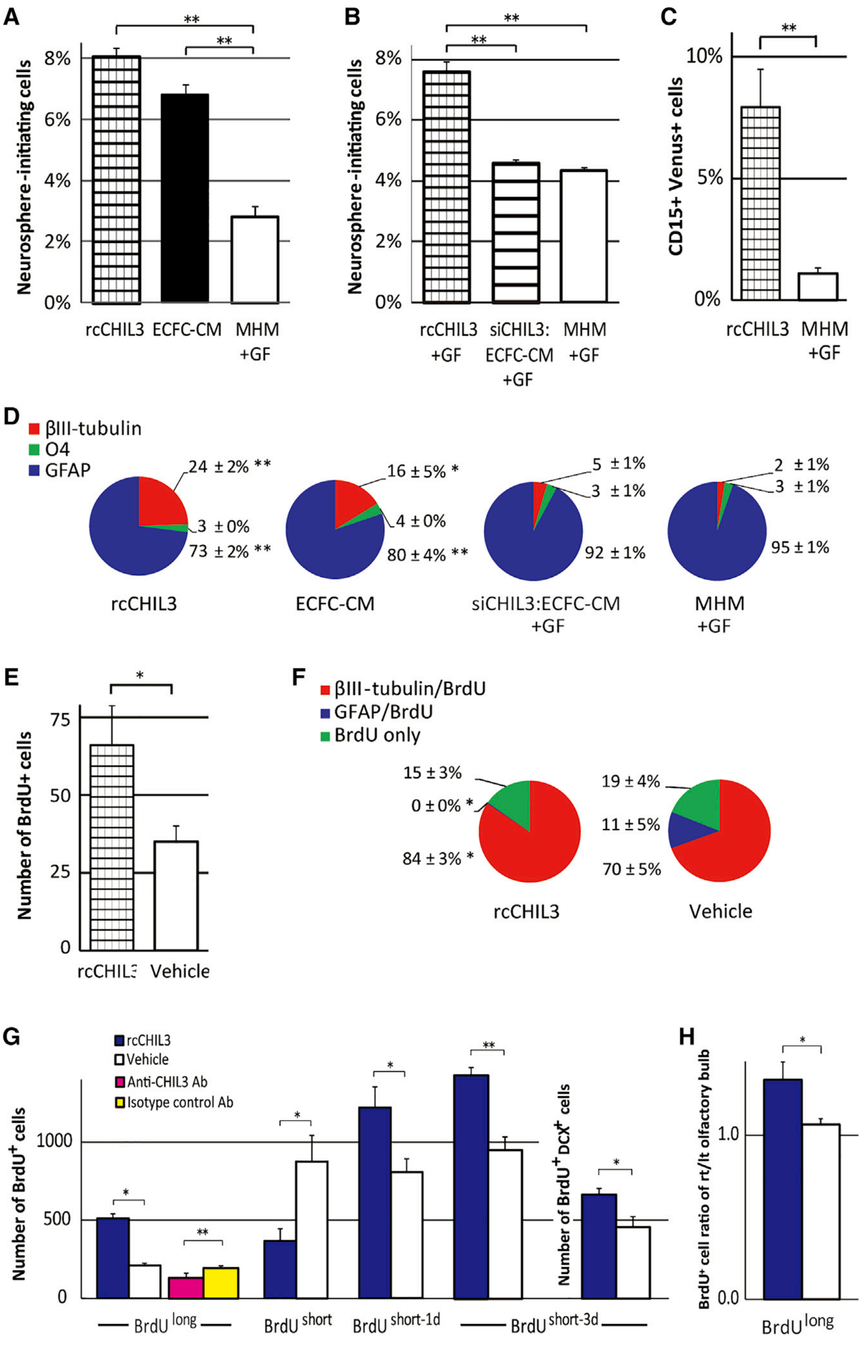

Figure 3. CHIL3 promotes NSC self-renewal and neurogenesis *in vitro* and *in vivo*

(A and B) Percentages of neurosphere-initiating cells in groups treated with recombinant CHIL3 (rcCHIL3), ECFC-CM, or control medium with GF (MHM + GF) (A; data of adult NSCs are provided in Figure S4B) as well as with rcCHIL3 + GF, *Chil3* knockdown ECFC-CM + GF (siChil3:ECFC-CM + GF), or MHM + GF (B). See also Figure S7 for the culture protocol. 18 or 20 (A) and 10 (B) independent experiments.

(C) Flow cytometry analysis of CD15- and Venus-positive cells in the population of rcCHIL3-treated or MHM + GF-treated neurospheres derived from E/nestin:dVenus transgenic mouse embryos. See also Figure S4C for plots. 3 independent experiments.

(D) Percentages of neurons (βIII-tubulin), oligodendrocytes (O4), or astrocytes (GFAP) after differentiation of neurospheres treated with rcCHIL3, ECFC-CM, siChil3:ECFC-CM + GF, or MHM + GF. See also Figure S4D for triple labeling images. 8 independent experiments.

(E and F) *Ex vivo* differentiation of V-SVZ NSCs obtained from mice infused with rcCHIL3 or vehicle. V-SVZ cells were dissected after infusion and cultured to differentiate with BrdU. BrdU-positive (E), βIII-tubulin/BrdU double-positive, or GFAP/BrdU double-positive cells (F) were counted. See also Figure S4E for images of βIII-tubulin/BrdU double-positive cells. 5 independent experiments.

(G) BrdU-positive cell counts in the V-SVZ after ventricular infusion of rcCHIL3, vehicle, anti-CHIL3 neutralizing antibodies, or isotype control antibodies. Mice were assigned to distinct regimens of BrdU administration as shown in Figure 1A. Three days after short-term labeling ($BrdU^{short-3d}$), BrdU/DCX double-positive cells in the V-SVZ were counted. 3–7 independent experiments for each group.

(H) BrdU-positive cells in the granule cell layer of the olfactory bulb were expressed as ratios of the count at the injected side to that at the contralateral side. Mice were administered rcCHIL3 or vehicle and subjected to $BrdU^{long}$. 2 or 3 independent experiments. *$p < 0.05$, **$p < 0.01$ (D and F, Student's t test compared with the MHM + GF-treated group).

lot of VEGFR2-positive ECs (Figure 4G). CHIL3 was distributed close to VEGFR2-positive ECs rather than VEGFR1 single-positive vessels (Figure 4H). CD31-positive microvessels expressed CHIL3, especially at sites in contact with SVZ GFAP-positive NSCs (Figure 4I). CHIL3-expressing SVZ microvessels did not express Aquaporin-4 (AQP4) (Figure 4J). This was consistent with the results of a previous study, which reported that the SVZ vasculature is in direct contact with NSCs at sites lacking a covering of astrocyte endfeet (Tavazoie et al., 2008). The specialized microanatomy and CHIL3 expression patterns of the SVZ vessels indicate that the CHIL3 signal diffuses from the microvessels to

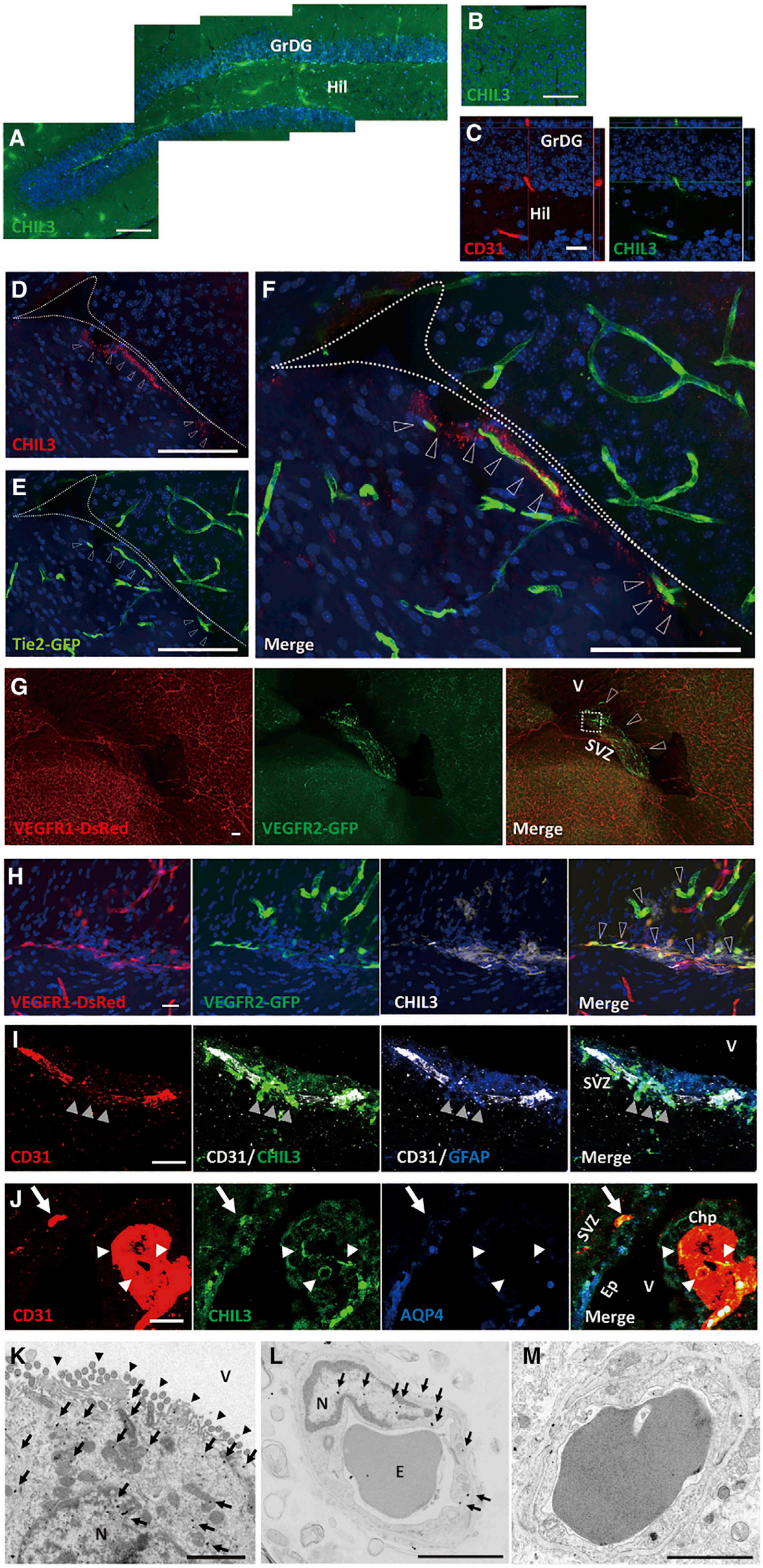

Figure 4. CHIL3 is endogenously expressed in the SGZ of the hippocampus and the V-SVZ

(A) Coronal views of the SGZ, the border between the granular layer of the dentate gyrus (GrDG), and the hilus (Hil).
(B) Negative for CHIL3 in the cortex.
(C) Confocal images demonstrating that CD31-positive ECs co-express CHIL3 in the SGZ.
(D–F) Coronal views of z stack confocal images of a Tie2-GFP transgenic mouse, showing the distribution of endogenous CHIL3. Dotted lines indicate the lateral ventricle. CHIL3 expression was marked in the vicinity of Tie2-positive ECs in the SVZ of the lateral side of the ventricle (open arrowheads).
(G) Sagittal images of the forebrain of an Flk1-GFP BAC; Flt1-tdsRed BAC double transgenic mouse. VEGFR2 was exclusively expressed in the V-SVZ (open arrowheads).
(H) VEGFR1, VEGFR2, and CHIL3 expression in SVZ microvessels of an Flk1-GFP BAC; Flt1-tdsRed BAC double-transgenic mouse. High magnifications of a rectangle in panel G demonstrate the closeness of the distributions of CHIL3 and VEGFR2-positive ECs (open arrowheads).
(I) CD31-positive ECs, GFAP-positive NSCs, and CHIL3 expression in the V-SVZ. V-SVZ NSCs are in contact with ECs expressing CHIL3 (arrowheads).
(J) CD31-positive ECs and CHIL3 and AQP4 expression in the V-SVZ. Vessels of the SVZ (arrows) and the Chp (arrowheads) lack the AQP4 covering and express CHIL3. The AQP4-positive ependymal layer also expresses CHIL3. See also Figure S5A for CHIL3 expression in S100β-positive ependymal cells.
(K–M) Immunoelectron micrographs of a ciliated (arrowhead) ependymal cell (K), an SVZ microvessel (L), and a cortical microvessel encompassing an erythrocyte (M). CHIL3-labeled nanogold particles are shown as black dots (arrows in K and L). Chp, choroid plexus; Ep, ependymal layer; E, erythrocyte; N, nucleus; V, lateral ventricle.
Scale bars, 100 μm (A, B, and D–G), 20 μm (C and H–J), and 2 μm (K–M).

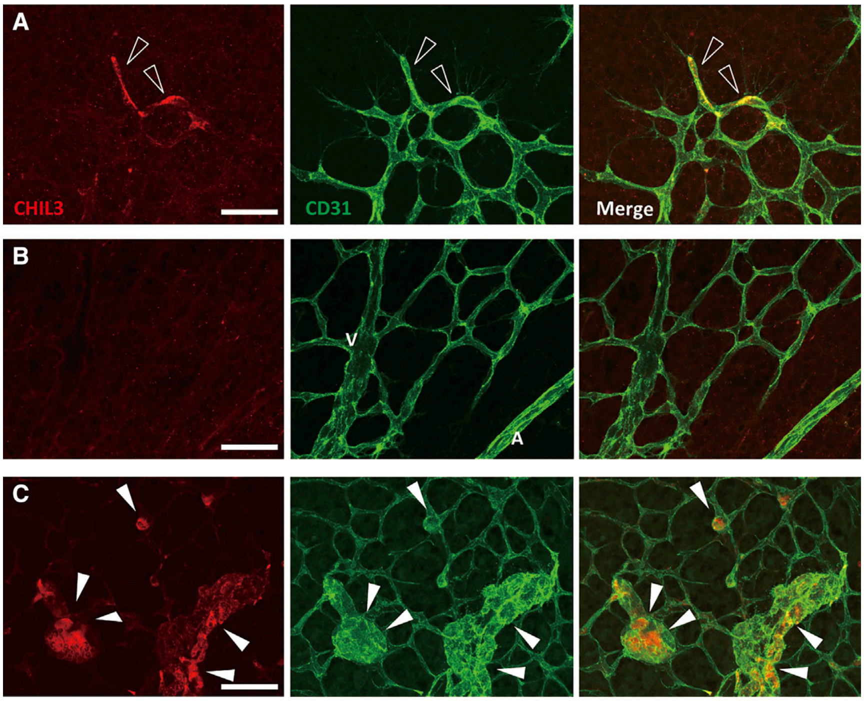

Figure 5. Retinal neovascular cells express CHIL3

(A–C) z stack confocal images showing CHIL3 expression and CD31-positive vasculature of post-natal day 5 (A), adult (B), and oxygen-induced retinopathy model (C) mouse retinas. CHIL3 is expressed in sprouting endothelial tip cells (open arrowheads in A) and pathological neovascular tufts (arrowheads in C) but not in mature ECs (B). See also Figure S5B for CHIL3 expression patterns in CD31-positive ECs in the injured brain. A, artery; V, vein. Scale bars, 50 μm.

NSCs. In addition to the SVZ microvessels, ECs in the choroid plexus (Chp) of the lateral ventricle did not exhibit AQP4 expression but did exhibit CHIL3 expression (Figure 4J). Because Chp contributes to production of CSF, CHIL3 may be secreted into the CSF. Marked amounts of CHIL3 were detected in crude CSF (48.3 ng/mL on average; Figure 2J). Another specialized structure of V-SVZ NSCs extends a minute apical ending that comprises a cilium at the ventricle surface (reviewed in Obernier and Alvarez-Buylla, 2019), which suggests that NSCs receive the CHIL3 signal from the ventricular CSF. Expression of CHIL3 was also detected in ependymal cells lining the wall of the ventricle (Figures 4J and S5A). Therefore, VEGFR2-positive ECs in the SVZ, CSF in the ventricle, and ventricular ependyma may provide the CHIL3 signal to NSCs in the V-SVZ NSC niche. Immunoelectron microscopy analysis confirmed CHIL3 expression in multi-ciliated ependymal cells and ECs of SVZ microvessels but not in cortical microvessels (Figures 4K–4M).

Endogenous expression of CHIL3 was also examined in the retina, another neurogenic niche that has been well studied for postnatal formation of blood vessels and pathological angiogenesis. Endothelial tip cells lead vascular sprouts at the tips of blood vessels and do not proliferate, whereas the neighboring stalk cells proliferate and form the vascular lumen (Siemerink et al., 2013). CHIL3 was expressed in tip cells but not in mature vessels (Figures 5A and 5B). Tip cells predominantly express *Vegfr2* rather than *Vegfr1* (Jakobsson et al., 2010). Thus, CHIL3-expressing ECs in the SVZ and the retina were not proliferative and expressed VEGFR2. In an ischemic retinopathy model, abnormally proliferated ECs (neovascular tufts) exhibited marked expression of CHIL3 (Figure 5C). Pathological CHIL3 expression was also observed in injury-related neovascularization of the brain (Figure S5B).

rcCHIL3 induces specific gene expression and protein phosphorylation in NSCs

To explore the intracellular responses elicited by CHIL3 in NSCs, gene expression was analyzed using a microarray. Cluster analysis revealed that rcCHIL3 upregulated cluster 1 gene expression as early as at 3 days and downregulated by 7 days compared with those in the groups treated with MHM + GF for 3 and 7 days (Figure 6A). The expression levels of cluster 2 genes were specifically upregulated at 7 days by rcCHIL3. Cluster 1 comprised canonical pathway-related genes associated with embryonic stem cell pluripotency, axon guidance signaling, vitamin D receptor, and retinoid X receptor activation (the regulation of NSC proliferation and differentiation is reviewed in Cui et al., 2017) or factors promoting cardiogenesis (Figure S6A). Cluster 1 genes included 10 titles of genes related to the maintenance of neural stem/progenitor cells or neurogenesis according to the annotation of the protein database Swiss-Prot or publications (Figure 6D). For example, the cluster 1 gene *Tgfb2*, which is reported to be a Chp-secreted factor, promotes NSC colony formation (Silva-Vargas et al., 2016). *Nog*, a cluster 1 gene reported to play a

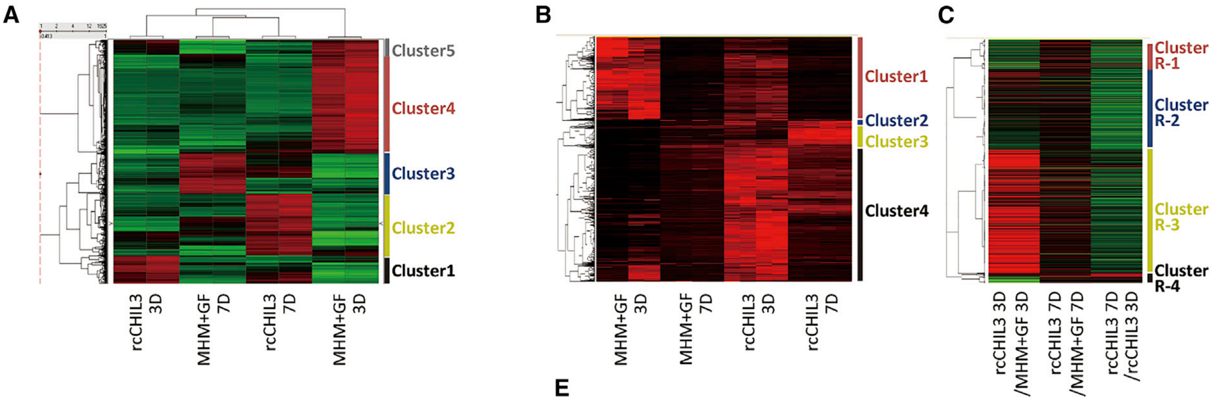

D

**Early expression genes**	rcCHIL3 exposure 3D	7D	Gene Title (in order of expression levels compared to MHM+GF at 3D)	Gene Symbol
Expression pattern: Cluster1	↘			
Expression ratio to MHM+GF	++	++~+	semaphorin 3B	Sema3b
	++	++~+	ephrin A1	Efna1
	++	++~+	S100 protein, beta polypeptide, neural	S100b
	++	++~+	transforming growth factor, beta 2	Tgfb2
	++	++~+	bone morphogenetic protein 4	Bmp4
	++	++~+	contactin 3	Cntn3
	++	—	doublecortin	Dcx
	++	—	noggin	Nog
	+	—	NK2 transcription factor related, locus 2	Nkx2-2
	+	—	hairy and enhancer of split 5	Hes5

E

**Early phosphorylation proteins**	rcCHIL3 exposure 3D	7D	Protein (in order of phosphorylation levels compared to MHM+GF at 3D)	AccNum
Phosphorylation pattern: Cluster4	↘			
Phosphorylation ratio to MHM+GF: Cluster R-3	++	++~+	DNA-binding protein inhibitor ID-2	ID2_MOUSE
	++	++~+	Microtubule-associated protein 2	MAP2_MOUSE
	++	++~+	MAP1 light chain LC1	MAP1B_MOUSE
	++	++~+	Netrin-3	NET3_MOUSE
	++	++~+	Neuronal migration protein doublecortin	DCX_MOUSE
	++	++~+	Plexin-B3	PLXB3_MOUSE
	++	++~+	Transcription factor SOX-8	SOX8_MOUSE
	++	++~+	Contactin-5	CNTN5_RAT
	++	++~+	Glial fibrillary acidic protein	GFAP_MOUSE
	++	++~+	Fibroblast growth factor receptor 4	FGFR4_RAT
	++	++~+	Transcription factor SOX-6	SOX6_MOUSE
	++	++~+	Neural cell adhesion molecule 1	NCAM1_MOUSE
	++	++~+	Catenin beta-1	CTNB1_MOUSE
	++	—	Transcription activator BRG1	SMCA4_MOUSE
	++	—	Ephrin type-B receptor 2	EPHB2_MOUSE
	++	—	Tubulin beta-2B chain	TBB2B_MOUSE
	++	—	Nestin	NEST_MOUSE
	++	—	Plexin-C1	PLXC1_MOUSE

F

**Early and increasing expression genes**	rcCHIL3 exposure 3D	7D	Gene Title (in order of expression levels compared to MHM+GF at 3D)	Gene Symbol
Expression pattern: Cluster2	↗			
Expression ratio to MHM+GF	++	+	glial fibrillary acidic protein	Gfap
	++	+	chemokine (C-X-C motif) receptor 4	Cxcr4
	++	+	ATPase, Na+/K+ transporting, alpha 1 polypeptide	Atp1a1
	—~+	++	semaphorin 3D	Sema3d
	—~+	++	meteorin, glial cell differentiation regulator	Metrn
	—~+	++	vesicle-associated membrane protein 5	Vamp5
	—~+	++	secreted phosphoprotein 1	Spp1

G

**Early and increasing phosphorylation proteins**	rcCHIL3 exposure 3D	7D	Protein (in order of phosphorylation levels compared to MHM+GF at 3D)	AccNum
Phosphorylation pattern: Cluster3	↗			
Phosphorylation ratio to MHM+GF: Cluster R-3	++	++	Glial fibrillary acidic protein	GFAP_MOUSE
	++	++	Numb-like protein	NUMB_MOUSE

Figure 6. CHIL3 induces specific gene expression and protein phosphorylation in NSCs

(A–C) Cluster analyses of gene expression levels (A; red, upregulated expression; green, downregulated expression), protein phosphorylation levels (B; red, higher levels of phosphorylation; dark red, lower levels of phosphorylation), and the ratio of phosphorylation levels between samples (C; red and green indicate the ratio of phosphorylation levels of >1 and <1, respectively) in rcCHIL3-treated NSCs relative to those in NSCs cultured with the control medium supplemented with GF (MHM + GF). NSCs cultured for 3 days or 7 days were subjected to microarray or phosphoproteome analysis (2 for each). See also Figure S7 for the culture protocol. Canonical pathways of the cluster 1 genes in (A) are provided in Figure S6A.

(D–G) rcCHIL3-induced gene expression and protein phosphorylation associated with maintenance of neural stem/progenitor cells or neurogenesis in NSCs. CHIL3-induced responses are characterized as “early” (D and E) and “early and increasing” (F and G). The ratios of gene expression (D and F) and protein phosphorylation levels (E and G) in the rcCHIL3-treated groups to those in the MHM + GF-treated group of >2, 1–2, and 0–1 are designated as ++, +, and —, respectively.

Complete lists of the genes, proteins with phosphorylated sites, and the results of cluster analysis are provided in Data S1 and S2.

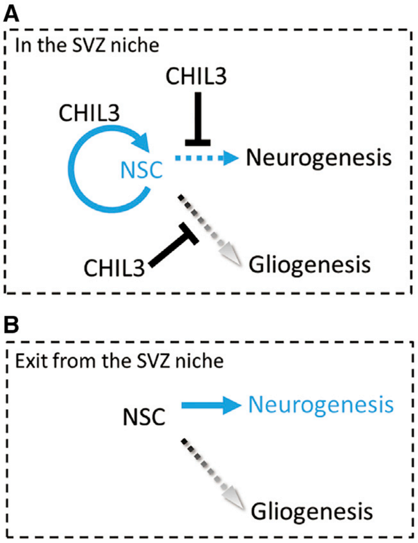

Figure 7. Schematic of the role of CHIL3 in the V-SVZ niche
(A) CHIL3 promotes NSC self-renewal in the V-SVZ niche. Multipotent NSCs commit to neurogenesis rather than gliogenesis.
(B) NSCs migrate from the niche with the CHIL3 signal and predominantly differentiate into neurons through transit-amplifying cells and neuroblasts.

major role in the neurogenic microenvironment of the brain (Lim et al., 2000), was examined in this study as a representative gene induced by CHIL3 in NSCs. Quantitative reverse-transcriptase polymerase chain reaction (qRT-PCR) analysis revealed that the expression of *Nog* in the group treated with rcCHIL3 for 3 days was more than 10 times higher than that in the group treated with MHM + GF for 3 days (Figure S6B). Expression of *Nog* was downregulated in the group treated with rcCHIL3 for 7 days, which is consistent with the expression pattern of cluster 1 genes. Immunostaining of neurospheres revealed that synthesis and secretion of Noggin on day 3 after rcCHIL3 treatment were prominent compared with those on day 3 after MHM + GF treatment, and high expression was observed for at least 7 days (Figure S6C). Cluster 2 genes that were associated with maintenance of neural stem/progenitor cells or neurogenesis comprised seven titles of genes, including *Spp1*, which has been proposed as a novel Chp factor for NSC activation (Silva-Vargas et al., 2016; Figure 6F). The complete gene list is shown in Data S1.

The levels of phosphorylated proteins, which indicate functional activation of target proteins, in rcCHIL3-treated NSCs were examined. Cluster analysis revealed that rcCHIL3 induced early phosphorylation of cluster 4 proteins on day 3 (Figure 6B). The phosphorylation levels of cluster 3 proteins were increased on day 7 by rcCHIL3 treatment. Cluster analysis of the ratio also revealed that cluster R-3 proteins were markedly phosphorylated by rcCHIL3 on day 3 compared with those in the MHM + GF-treated group (rcCHIL3/MHM + GF on day 3 > 1) (Figure 6C). Nineteen proteins related to maintenance of neural stem/progenitor cells or neurogenesis were classified into cluster R-3 (Figures 6E and 6G). The complete list of phosphorylated proteins is shown in Data S2.

DISCUSSION

This study demonstrated that CHIL3 is a novel niche factor for NSCs. The findings of this study and specialized microanatomy of the adult mammalian V-SVZ suggest that NSCs receive the CHIL3 signal through direct contact with the AQP4-deficient SVZ vasculature and from the CSF through an apical ending to the ventricle. Thus, CHIL3 is a key niche factor for NSCs and promotes NSC expansion in the V-SVZ and NSC neurogenesis after NSCs migrate from the V-SVZ (for a presumptive mechanism, see Figure 7). CHIL3 is considered an intrinsic factor for the NSC niche because of its endogenous expression and distribution in the CSF. The principal sources of CHIL3 were VEGFR2-positive ECs of the microvasculature, ependymal cells of the ventricular wall, and Chp in the ventricle. Chp, which produces CSF, has been proposed as an NSC niche compartment that secretes novel candidate factors (Silva-Vargas et al., 2016). However, CHIL3 was not previously identified as a secretory protein. Preliminary data revealed expression of CHIL3 in endothelial tip cells of the developing retina and the serum, which indicated that CHIL3 is a critical neurogenic niche factor outside of the brain as well.

The amino acid sequence of CHIL3 (also called ECF-L or Ym1) is highly conserved in the chitinase family, but CHIL3 does not exhibit chitinase activity (Bussink et al., 2007). In the CNS, CHIL3 is expressed in microglial cells (Ponomarev et al., 2007) and has been reported recently to induce NSCs to activate oligodendrogenesis in the V-SVZ of the experimental autoimmune encephalomyelitis model (Starossom et al., 2019). This discrepancy between promotion of neurogenesis and oligodendrogenesis can be attributed to the difference between non-pathological and inflammatory models and the different *in vitro* experimental protocols. Neural stem/progenitor cells are exposed to various signals in the niche microenvironment, including the CHIL3 signal. The migration of NSCs from the V-SVZ niche promotes their differentiation because of the absence of the CHIL3 signal. To mimic the dynamics of *in vivo* fate, NSCs were cultured with rcCHIL3, and the rcCHIL3-treated NSCs were allowed to differentiate in the absence of rcCHIL3 (see culture protocol in Figure S7). The results indicate that CHIL3 recruits neuronal lineage-committed neural stem/progenitor cells in the V-SVZ niche rather than supporting NSC maturation into the neuronal

lineage. In contrast to the findings of this study, CHIL3-mediated oligodendrogenesis was observed in medium supplemented with CHIL3 (Starossom et al., 2019). A plausible explanation for this observation is that CHIL3 induces neuronal fate in the V-SVZ NSC niche but mediates oligodendrogenesis at inflammatory lesions away from the niche. In this study, the results of experiments with a cold injury model revealed CHIL3 expression in the vasculature of the brain lesion (Figure S5E). Ectopic expression of CHIL3 in various CNS disorders is expected to promote tissue repair. For example, chitinase-3-like protein 1 (CHI3L1), a human paralog of mouse CHIL3, has been widely studied as a biomarker of disease progression and tissue damage in multiple sclerosis (Burman et al., 2016), although its functions in the CNS remains unclear. Injury-related CHIL3 expression was detected in the experimental model of the cold-injured brain (Figure S5B) and ischemic retinopathy (Figure 5C). In humans, CHI3L1 signaling also plays a critical role in cancer and is increasingly applied as a therapeutic target for various malignancies, including glioma and colon cancer (Zhao et al., 2020). Further studies using mouse models or human/patient-derived induced pluripotent stem cells are needed to examine whether mouse CHIL3 and human CHI3L1 are involved in tissue repair in diseases of the CNS and other organs.

The *in vivo* infusion and *in vitro* neurosphere assays demonstrated that CHIL3 induced NSC expansion by promoting NSC self-renewal. NSCs have been reported to undergo slow division. Hence, expansion of NSCs is unlikely to enhance BrdU incorporation into neurospheres when cultured with BrdU *in vitro* and requires the $\mathrm{BrdU^{long}}$ regimen for *in vivo* visualization (see also Figure 1). The results of the neurosphere assay revealed the self-renewal capacity of rcCHIL3-treated neurospheres that were passaged in medium without rcCHIL3 (expressed as the number of tertiary neurospheres generated from neurosphere-initiating cells in rcCHIL3-treated secondary neurospheres; see culture protocol in Figure S7). The results of *in vivo* studies indicate that CHIL3 suppresses generation of transit-amplifying cells from NSCs (see the proposed model of CHIL3 functions in Figures 1B and 7A). Thus, the *in vitro* and *in vivo* data indicate that CHIL3 maintains NSCs with self-renewal capacity and promotes their commitment to the neuronal lineage in the neurogenic niche. Because we detected endogenous expression of CHIL3 in the SGZ of the hippocampus and retina, additional studies focused on these regions are expected to further reveal the biological effects of CHIL3 in neurogenic regions other than the SVZ.

Among approximately 2,000 differential proteins between ECFC-CM- and MSC-CM-specific spots identified in this study using differential proteomics analysis, the top 14 ECFC-CM-specific spots were subjected to MS and searched against the protein database. CHIL3, PEDF, and Gelsolin were identified, but the remaining four candidates could not be matched to the database, except for serotransferrin derived from serum in the medium (Figure 2G). EC-secreted soluble factors have been reported to modulate NSC activity and regulate NSC self-renewal (Shen et al., 2004). In particular, PEDF has been proposed as a candidate signal (Ramirez-Castillejo et al., 2006). PEDF alone could not sustain neurosphere formation in the absence of FGF-2 or EGF, whereas CHIL3 sustained neurosphere formation in medium not supplemented with FGF-2 or EGF and potentiated GF-induced generation of neurospheres. Further studies are needed to examine the synergistic activities of CHIL3 and PEDF as NSC niche factors. Gelsolin, which has been identified as an ECFC-specific protein in this study, is an actin-binding protein that has been reported to play a critical role in NSC migration (Kronenberg et al., 2010) and can be detected in the CSF (Lind et al., 2016). This study demonstrated that CHIL3 solely maintained NSCs with neuronal commitment. However, it is plausible that CHIL3 and several factors, including PEDF, Gelsolin, known GFs, and unknown proteins not identified in the differential proteomics analysis, synergistically maintain the neurogenic niche microenvironment.

The crystal structure of CHIL3 comprises a saccharide-binding site (Sun et al., 2001). Functional studies have demonstrated that CHIL3 does not bind to chitin (an oligomer of N-acetylglucosamine) but binds to saccharides with a free amine group, such as glucosamine and its oligomers (Chang et al., 2001). Transcriptome and phosphoproteome analyses did not reveal specific intracellular signaling of NSCs induced by CHIL3 but showed that CHIL3 activated large numbers of genes and proteins associated with maintenance of neural stem/progenitor cells or neurogenesis. CHIL3 is considered an EGFR ligand or a co-ligand because it induces the signaling molecules of the mitogen-activated protein kinase (MAPK) pathway to promote phosphorylation (Starossom et al., 2019). EGFR has an *N*-glycosylated extracellular domain. Interleukin-13 receptor subunit alpha-2, transmembrane protein 219, galectin-3, and CD44 have been identified as receptors of human CHI3L1 (reviewed in Zhao et al., 2020). The findings of this study suggest that CHIL3 may act as a positive modulator through activation of multiple molecules related to NSC self-renewal and neuronal commitment rather than targeting a specific intracellular signaling pathway. Development and phenotyping of a CHIL3 knockout or conditional knockout mouse model may elucidate the mechanisms underlying CHIL3 signaling.

Numb promotes neurogenesis by inhibiting the Notch pathway. This study demonstrated that CHIL3 elicited

early and increased phosphorylation of Numb (cluster 3 and cluster R-3 in Figures 6B, 6C, and 6G). The phosphorylated site was S295 (Data S2), which corresponded with Numb phosphorylation at serine 7 and 295, involved in regulation of the polarity of radial glial cells during mammalian development (Smith et al., 2007). Phosphorylation is required to restrict Numb localization to the lateral membrane of polarized cells, which downregulates the levels of active Numb and, consequently, maintains radial glial cells in their progenitor state by activating Notch (Lui et al., 2011). In contrast, a daughter cell exhibits upregulated levels of active Numb, which leads to inhibition of Notch signaling and enhances neuronal differentiation. This mechanism of Numb phosphorylation is presumptive to the CHIL3 niche signal that maintains NSCs in the V-SVZ. NSCs undergo neurogenesis when they migrate from the V-SVZ niche (see also Figure 7). This asymmetric localization of Numb has been observed not only in dividing cells of the brain but also in progenitors of the retina (Kechad et al., 2012). In this study, expression of CHIL3 was detected in neovascular cells of the retina (Figure 5).

Using differential proteomics analysis, this study identified CHIL3 as an ECFC-specific secreted factor along with PEDF and Gelsolin. CHIL3 was demonstrated to be a novel niche factor for NSCs because it promoted NSC self-renewal and commitment to the neuronal lineage. Endogenous expression of CHIL3 was observed in the neurogenic niche of the brain and retina. In the V-SVZ of the brain, specialized VEGFR2-positive ECs, ependymal cells of the ventricular wall, and the Chp in the ventricle comprised the niche microenvironment of CHIL3. A diverse repertoire of intracellular factors mediated by CHIL3 suggests spatiotemporal regulation of NSCs in the V-SVZ niche. The molecular mechanisms of CHIL3 must be elucidated by investigating temporal subpopulations of NSCs and their progeny (quiescent NSCs, activated NSCs, transit-amplifying cells, and neuroblasts) (Codega et al., 2014; Pastrana et al., 2009).

EXPERIMENTAL PROCEDURES

A detailed description of materials and methods can be found in the supplemental experimental procedures.

Resource availability

Corresponding author

Requests for resources, reagents, and protocols should be addressed to and will be fulfilled by the corresponding author, J.N. (namiki@med.keio.ac.jp).

Materials availability

Unique reagents generated in this study are available upon request from the corresponding author.

Animals

All animal-related procedures were approved by the Laboratory Animal Care and Use Committee of Keio University and were conducted in accordance with the guidelines of the National Institutes of Health. Adult (8- to 10-week-old) wild-type C57BL/6J mice, wild-type ICR mouse embryos, and adult CAG-EGFP transgenic mice were purchased from SLC (Shizuoka, Japan). E/nestin:dVenus transgenic mouse embryos (Sunabori et al., 2008) were bred in the animal facility at Keio University School of Medicine (Tokyo, Japan). Adult Tie2-GFP transgenic mice (The Jackson Laboratory, Bar Harbor, ME, USA) were obtained courtesy of H. Toriumi (Neurology, Keio University School of Medicine). Adult Flk1-GFP BAC; Flt1-tdsRed BAC double-transgenic mice (Matsumoto et al., 2012) were established and provided by M. Ema (University of Tsukuba, Ibaraki, Japan).

Cell culture and CM

ECFCs were established under adherent culture conditions for 21 days *in vitro* (DIV) from adult murine bone marrow mononuclear cells (Suzuki et al., 2010). ECFCs were lifted and cultured for another 14 DIV to obtain committed mature ECs. Aliquots of ECFCs and mature ECs were processed for validation of the immunocytochemical phenotypes. A mouse brain endothelioma cell line (bEnd.3 cells, CRL-2299, American Type Culture Collection, Manassas, VA, USA) were cultured according to the manufacturer's instructions. MSCs were isolated from whole bone marrow cells of adult mice by their adherence to plastic. Neurospheres were generated from embryonic day 14 or adult mouse forebrain striatum cells by the floating culture method in control medium of MHM supplemented with FGF-2 and EGF, as described previously with some modifications (Kase et al., 2019).

At the end of the culture period, ECFCs, mature ECs, EC lines, and MSCs were washed and cultured with MHM for another 1 DIV. CM was then collected and filtered. For immunoblot analysis, MHM was made without transferrin. For immunoblotting and *in vivo* intraventricle infusion, CM were concentrated with Amicon Ultra centrifugal filter devices (UFC9 010, UFC8 010, Millipore) at 2,380 × g for 30 min. Sample volumes were recovered at approximately 45-fold concentration.

Immunostaining

Adult mouse forebrains were cut coronally through the anterior part of the lateral ventricles (from the level of the bregma to 1.2 mm rostral) and the olfactory bulb. Eyes isolated from mice were prepared for whole-mount samples. Indirect immunofluorescence was carried out with standard protocols and in more than two independent experiments to confirm immunoreactivity.

Differential proteomics

Proteins were extracted from ECFC-CM and MSC-CM after removal of albumin, immunoglobulin G (IgG), and transferrin. Samples were labeled with fluorescence and processed for 2D-DIGE. Proteins were separated according to isoelectronic point and mass. After spot detection and statistical analysis, spots of interest were picked from the ECFC-CM gel, digested, and analyzed via nanoLC-MS/MS. The protein databases Swiss-Prot and NCBInr were searched using the nanoLC-MS/MS data with a Mascot search engine (Matrix Science, Boston, MA, USA).

rcCHIL3 and *Chil3* knockdown

rcCHIL3 was produced from HEK293T cells that were transfected with the gene encoding *Chil3* using standard protocols. For *Chil3* knockdown experiments, *Chil3* siRNA was prepared by the Sigma Genosys siRNA Service (Sigma-Aldrich) and designed as follows: sense strand, 5′-GAUCAAGUUCAACGGUUUUUC-3′, antisense strand, 5′-AAAACCGUUGAACUUGAUCUU-3′. The optimal concentration of *Chil3* siRNA to effectively deplete CHIL3 in ECFC-CM was determined (Figure S4A). Cells transfected with *Chil3* DNA or *Chil3* siRNA were washed and cultured with MHM for another 1 DIV. CM of CHIL3-expressing HEK293T (rcCHIL3) or CHIL3-depleted ECFCs (siChil3:ECFC-CM) was then collected.

Microarray analysis and qRT-PCR

Secondary neurospheres were cultured with rcCHIL3 or MHM + GF for 3 or 7 DIV (Figure S7). Total RNA was isolated from neurospheres and applied to DNA microarray analysis using Affymetrix Gene Chip technology. Total RNA isolated from neurospheres, V-SVZ cells, and heart muscle cells was also processed for qRT-PCR with the following *Nog* primers: forward, 5′-TGCTGTACGCGTGGAATGA-3′; reverse, 5′-TGAGG TGCACAGACTTGGATG-3′.

Quantitative phosphoproteome analysis

Proteins were extracted from secondary neurospheres cultured with rcCHIL3 or MHM + GF for 3 or 7 DIV and processed for phosphoproteome analysis based on nanoLC-MS/MS (Figure S7). Peptides and proteins were identified, and the peak areas of each phosphopeptide were subjected to cluster analysis.

In vivo intraventricle infusion and BrdU labeling

ECFC-CM, rcCHIL3, anti-CHIL3 antibody (rabbit polyclonal, 01404, STEMCELL Technologies, Vancouver, BC, Canada), isotype control antibody (rabbit polyclonal IgG, ab27472, Abcam, Cambridge, MA, USA), or vehicle (αMEM against ECFC-CM or MHM against rcCHIL3) was infused into the mouse right lateral ventricle for 7 days. rcCHIL3 and ECFC-CM were concentrated (8.9 μg/mL of rcCHIL3 on average, measured by ELISA, two independent experiments). Antibodies were purified with an antibody purification kit (ab102784, Abcam).

For $BrdU^{long}$, BrdU (B5002, Sigma-Aldrich) was administered to adult mice through their drinking water (1 mg/mL) for 14 days (Figure 1). After BrdU administration, the mice were allowed to survive another 7 days without BrdU. Adult mice in the $BrdU^{short}$ group were injected intraperitoneally with BrdU (50 mg/kg, 50 μL of 25 mg/mL) 3 h prior to sacrifice on the last day of the infusion. Mice in the $BrdU^{short\text{-}1d}$ or $BrdU^{short\text{-}3d}$ groups received BrdU 1 day or 3 days after termination of the infusion, respectively (Figures 1 and 3G).

Oxygen-induced retinopathy and cold injury of the brain

Post-natal day 8 mice with nursing mothers were maintained for 3 days in 85% oxygen and then placed back in room air with modification as described previously (Kubota et al., 2009). The established vasculature was obliterated by hyperoxic insult, resulting in an ischemic area. Subsequently, revascularization occurred to recover normal vasculature.

Cold injury was induced in the adult mouse forebrain as described previously (Suzuki et al., 2010). A 2-mm-diameter metal probe, cooled in liquid nitrogen, was placed through the hole drilled into the skull and applied to the dura mater for 30 s. Neovascularization by ECs expressing vascular Nestin was established at the injury site within 14 days.

Statistics

All results are expressed as mean ± SEM. Comparisons between the mean variables of 2 groups were made by 2-tailed Student's t test. p values less than 0.05 were considered to be statistically significant.

DATA AND CODE AVAILABILITY

The accession number for the microarray data in this paper is the GEO database (https://www.ncbi.nlm.nih.gov/geo/): GSE57794. The accession number for the phosphoproteome analyses in this paper is the ProteomeXchange Consortium (http://proteomecentral.proteomexchange.org) via the jPOST partner repository (https://jpostdb.org): PXD037411.

SUPPLEMENTAL INFORMATION

Supplemental information can be found online at https://doi.org/10.1016/j.stemcr.2022.10.012.

AUTHOR CONTRIBUTIONS

Conceptualization, J.N.; methodology, J.N., S. Suzuki, S. Shibata, Y.K., K.Y., Y.M., Y.I., K.S., and H.O.; investigation, J.N., S. Suzuki, S. Shibata, Y.K., N.K., K.Y., R.Y., and T.M.; manuscript preparation, J.N.; manuscript review and editing, Y.K., K.S., and H.O.; funding acquisition, J.N.

ACKNOWLEDGMENTS

We acknowledge the following colleagues for their assistance and scientific input: T. Shimazaki, S. Suzuki, S. Harigae, H. Kanki, T. Nagai (Keio University School of Medicine, Tokyo, Japan), H. Yamanaka (Chemicals Evaluation and Research Institute, Saitama, Japan), H. Ebise, and H. Nakagawa (Sumitomo Pharma Co. Ltd., Osaka, Japan). We thank T. Takahashi (Kyoto University, Kyoto, Japan) for reviewing the manuscript draft. J.N. was supported by Japan Society for the Promotion of Science KAKENHI (JP23122518 and JP25122715) and Keio Gijuku Academic Development Funds.

CONFLICT OF INTERESTS

The authors declare no competing interests.

Received: January 31, 2022
Revised: October 14, 2022
Accepted: October 17, 2022
Published: November 10, 2022

REFERENCES

Akter, M., Kaneko, N., and Sawamoto, K. (2021). Neurogenesis and neuronal migration in the postnatal ventricular-subventricular zone: similarities and dissimilarities between rodents and primates. Neurosci. Res. *167*, 64–69.

Burman, J., Raininko, R., Blennow, K., Zetterberg, H., Axelsson, M., and Malmeström, C. (2016). YKL-40 is a CSF biomarker of intrathecal inflammation in secondary progressive multiple sclerosis. J. Neuroimmunol. *292*, 52–57.

Bussink, A.P., Speijer, D., Aerts, J.M.F.G., and Boot, R.G. (2007). Evolution of mammalian chitinase(-like) members of family 18 glycosyl hydrolases. Genetics *177*, 959–970.

Castro-Garcia, P., Díaz-Moreno, M., Gil-Gas, C., Fernández-Gómez, F.J., Honrubia-Gómez, P., Álvarez-Simón, C.B., Sánchez-Sánchez, F., Cano, J.C.C., Almeida, F., Blanco, V., et al. (2015). Defects in subventricular zone pigmented epithelium-derived factor niche signaling in the senescence-accelerated mouse prone-8. Faseb. J. *29*, 1480–1492.

Chambers, S.E.J., Pathak, V., Pedrini, E., Soret, L., Gendron, N., Guerin, C.L., Stitt, A.W., Smadja, D.M., and Medina, R.J. (2021). Current concepts on endothelial stem cells definition, location, and markers. Stem Cells Transl. Med. *10* (*Suppl 2*), S54–S61.

Chang, N.C., Hung, S.I., Hwa, K.Y., Kato, I., Chen, J.E., Liu, C.H., and Chang, A.C. (2001). A macrophage protein, Ym1, transiently expressed during inflammation is a novel mammalian lectin. J. Biol. Chem. *276*, 17497–17506.

Codega, P., Silva-Vargas, V., Paul, A., Maldonado-Soto, A.R., Deleo, A.M., Pastrana, E., and Doetsch, F. (2014). Prospective identification and purification of quiescent adult neural stem cells from their in vivo niche. Neuron *82*, 545–559.

Critser, P.J., and Yoder, M.C. (2010). Endothelial colony-forming cell role in neoangiogenesis and tissue repair. Curr. Opin. Organ Transplant. *15*, 68–72.

Cui, X., Gooch, H., Petty, A., McGrath, J.J., and Eyles, D. (2017). Vitamin D and the brain: genomic and non-genomic actions. Mol. Cell. Endocrinol. *453*, 131–143.

Doetsch, F., Caillé, I., Lim, D.A., García-Verdugo, J.M., and Alvarez-Buylla, A. (1999). Subventricular zone astrocytes are neural stem cells in the adult mammalian brain. Cell *97*, 703–716.

Jakobsson, L., Franco, C.A., Bentley, K., Collins, R.T., Ponsioen, B., Aspalter, I.M., Rosewell, I., Busse, M., Thurston, G., Medvinsky, A., et al. (2010). Endothelial cells dynamically compete for the tip cell position during angiogenic sprouting. Nat. Cell Biol. *12*, 943–953.

Kase, Y., Otsu, K., Shimazaki, T., and Okano, H. (2019). Involvement of p38 in age-related decline in adult neurogenesis via modulation of wnt signaling. Stem Cell Rep. *12*, 1313–1328.

Kechad, A., Jolicoeur, C., Tufford, A., Mattar, P., Chow, R.W.Y., Harris, W.A., and Cayouette, M. (2012). Numb is required for the production of terminal asymmetric cell divisions in the developing mouse retina. J. Neurosci. *32*, 17197–17210.

Kronenberg, G., Gertz, K., Baldinger, T., Kirste, I., Eckart, S., Yildirim, F., Ji, S., Heuser, I., Schröck, H., Hörtnagl, H., et al. (2010). Impact of actin filament stabilization on adult hippocampal and olfactory bulb neurogenesis. J. Neurosci. *30*, 3419–3431.

Kubota, Y., Takubo, K., Shimizu, T., Ohno, H., Kishi, K., Shibuya, M., Saya, H., and Suda, T. (2009). M-CSF inhibition selectively targets pathological angiogenesis and lymphangiogenesis. J. Exp. Med. *206*, 1089–1102.

Lim, D.A., Tramontin, A.D., Trevejo, J.M., Herrera, D.G., García-Verdugo, J.M., and Alvarez-Buylla, A. (2000). Noggin antagonizes BMP signaling to create a niche for adult neurogenesis. Neuron *28*, 713–726.

Lind, A.L., Emami Khoonsari, P., Sjödin, M., Katila, L., Wetterhall, M., Gordh, T., and Kultima, K. (2016). Spinal cord stimulation alters protein levels in the cerebrospinal fluid of neuropathic pain patients: a proteomic mass spectrometric analysis. Neuromodulation *19*, 549–562.

Lui, J.H., Hansen, D.V., and Kriegstein, A.R. (2011). Development and evolution of the human neocortex. Cell *146*, 18–36.

Matsumoto, K., Azami, T., Otsu, A., Takase, H., Ishitobi, H., Tanaka, J., Miwa, Y., Takahashi, S., and Ema, M. (2012). Study of normal and pathological blood vessel morphogenesis in Flt1-tdsRed BAC Tg mice. Genesis *50*, 561–571.

Obernier, K., and Alvarez-Buylla, A. (2019). Neural stem cells: origin, heterogeneity and regulation in the adult mammalian brain. Development *146*, dev156059.

Palmer, T.D., Willhoite, A.R., and Gage, F.H. (2000). Vascular niche for adult hippocampal neurogenesis. J. Comp. Neurol. *425*, 479–494.

Pastrana, E., Cheng, L.C., and Doetsch, F. (2009). Simultaneous prospective purification of adult subventricular zone neural stem cells and their progeny. Proc. Natl. Acad. Sci. USA *106*, 6387–6392.

Ponomarev, E.D., Maresz, K., Tan, Y., and Dittel, B.N. (2007). CNS-derived interleukin-4 is essential for the regulation of autoimmune inflammation and induces a state of alternative activation in microglial cells. J. Neurosci. *27*, 10714–10721.

Ramírez-Castillejo, C., Sánchez-Sánchez, F., Andreu-Agulló, C., Ferrón, S.R., Aroca-Aguilar, J.D., Sánchez, P., Mira, H., Escribano, J., and Fariñas, I. (2006). Pigment epithelium-derived factor is a niche signal for neural stem cell renewal. Nat. Neurosci. *9*, 331–339.

Shen, Q., Goderie, S.K., Jin, L., Karanth, N., Sun, Y., Abramova, N., Vincent, P., Pumiglia, K., and Temple, S. (2004). Endothelial cells stimulate self-renewal and expand neurogenesis of neural stem cells. Science *304*, 1338–1340.

Shen, Q., Wang, Y., Kokovay, E., Lin, G., Chuang, S.M., Goderie, S.K., Roysam, B., and Temple, S. (2008). Adult SVZ stem cells lie in a vascular niche: a quantitative analysis of niche cell-cell interactions. Cell Stem Cell *3*, 289–300.

Siemerink, M.J., Klaassen, I., Van Noorden, C.J.F., and Schlingemann, R.O. (2013). Endothelial tip cells in ocular angiogenesis: potential target for anti-angiogenesis therapy. J. Histochem. Cytochem. *61*, 101–115.

Silva-Vargas, V., Maldonado-Soto, A.R., Mizrak, D., Codega, P., and Doetsch, F. (2016). Age-dependent niche signals from the choroid plexus regulate adult neural stem cells. Cell Stem Cell *19*, 643–652.

Smith, C.A., Lau, K.M., Rahmani, Z., Dho, S.E., Brothers, G., She, Y.M., Berry, D.M., Bonneil, E., Thibault, P., Schweisguth, F., et al. (2007). aPKC-mediated phosphorylation regulates asymmetric

membrane localization of the cell fate determinant Numb. EMBO J. *26*, 468–480.

Soares, R., Ribeiro, F.F., Lourenço, D.M., Rodrigues, R.S., Moreira, J.B., Sebastião, A.M., Morais, V.A., and Xapelli, S. (2021). The neurosphere assay: an effective in vitro technique to study neural stem cells. Neural Regen. Res. *16*, 2229–2231.

Starossom, S.C., Campo Garcia, J., Woelfle, T., Romero-Suarez, S., Olah, M., Watanabe, F., Cao, L., Yeste, A., Tukker, J.J., Quintana, F.J., et al. (2019). Chi3l3 induces oligodendrogenesis in an experimental model of autoimmune neuroinflammation. Nat. Commun. *10*, 217.

Sun, Y.J., Chang, N.C., Hung, S.I., Chang, A.C., Chou, C.C., and Hsiao, C.D. (2001). The crystal structure of a novel mammalian lectin, Ym1, suggests a saccharide binding site. J. Biol. Chem. *276*, 17507–17514.

Sunabori, T., Tokunaga, A., Nagai, T., Sawamoto, K., Okabe, M., Miyawaki, A., Matsuzaki, Y., Miyata, T., and Okano, H. (2008). Cell-cycle-specific nestin expression coordinates with morphological changes in embryonic cortical neural progenitors. J. Cell Sci. *121*, 1204–1212.

Suzuki, S., Namiki, J., Shibata, S., Mastuzaki, Y., and Okano, H. (2010). The neural stem/progenitor cell marker nestin is expressed in proliferative endothelial cells, but not in mature vasculature. J. Histochem. Cytochem. *58*, 721–730.

Tavazoie, M., Van der Veken, L., Silva-Vargas, V., Louissaint, M., Colonna, L., Zaidi, B., Garcia-Verdugo, J.M., and Doetsch, F. (2008). A specialized vascular niche for adult neural stem cells. Cell Stem Cell *3*, 279–288.

Zhao, T., Su, Z., Li, Y., Zhang, X., and You, Q. (2020). Chitinase-3 like-protein-1 function and its role in diseases. Signal Transduct. Targeted Ther. *5*, 201.

Stem Cell Reports, Volume *17*

Supplemental Information

Chitinase-like protein 3: A novel niche factor for mouse neural stem cells

Jun Namiki, Sayuri Suzuki, Shinsuke Shibata, Yoshiaki Kubota, Naoko Kaneko, Kenji Yoshida, Ryo Yamaguchi, Yumi Matsuzaki, Takeshi Masuda, Yasushi Ishihama, Kazunobu Sawamoto, and Hideyuki Okano

Figure S1

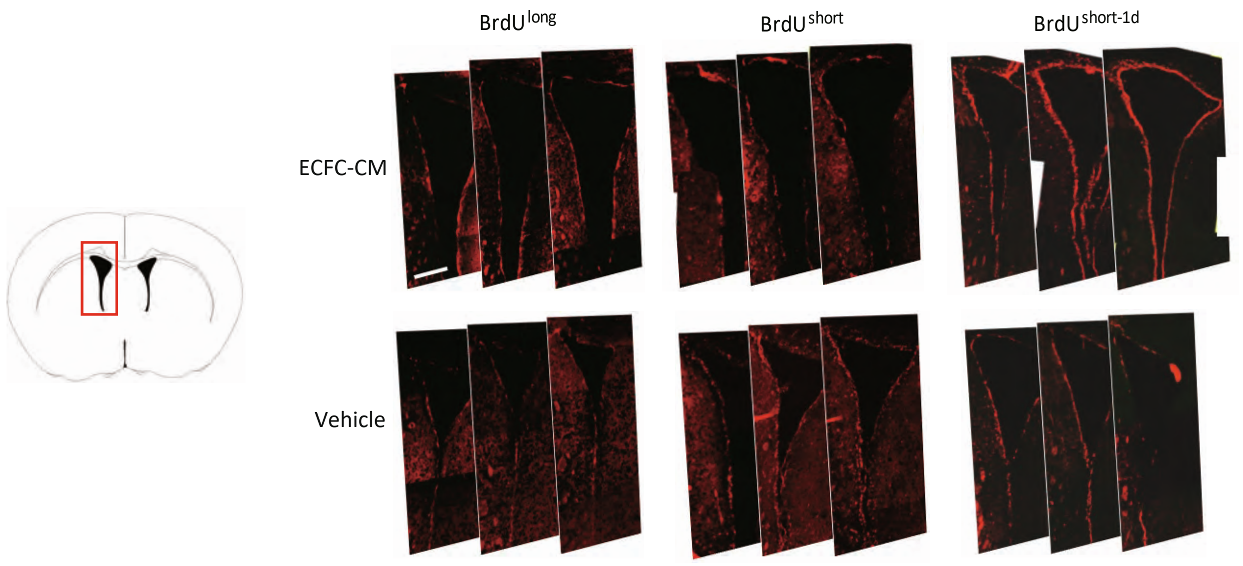

Figure S1. Immunostaining of BrdU-labeled cells (red) in the adult V-SVZ (related to Figure 1). ECFC-CM or vehicle was infused into the lateral ventricle for 7 days and assigned to distinct regimens of BrdU administration: $BrdU^{long}$, $BrdU^{short}$, or $BrdU^{short\text{-}1d}$. Coronal sections were prepared through the anterior part of the lateral ventricles. Representative immunofluorescence images of the ventricular zone (red square) were shown. Scale bar, 200 μm.

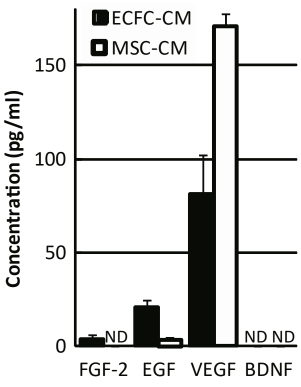

Figure S2. Concentrations of known growth factors in ECFC-CM and MSC-CM measured by ELISA (related to Figures 2B and 2C). ND, not detected. 2 or 3 independent experiments.

Figure S3

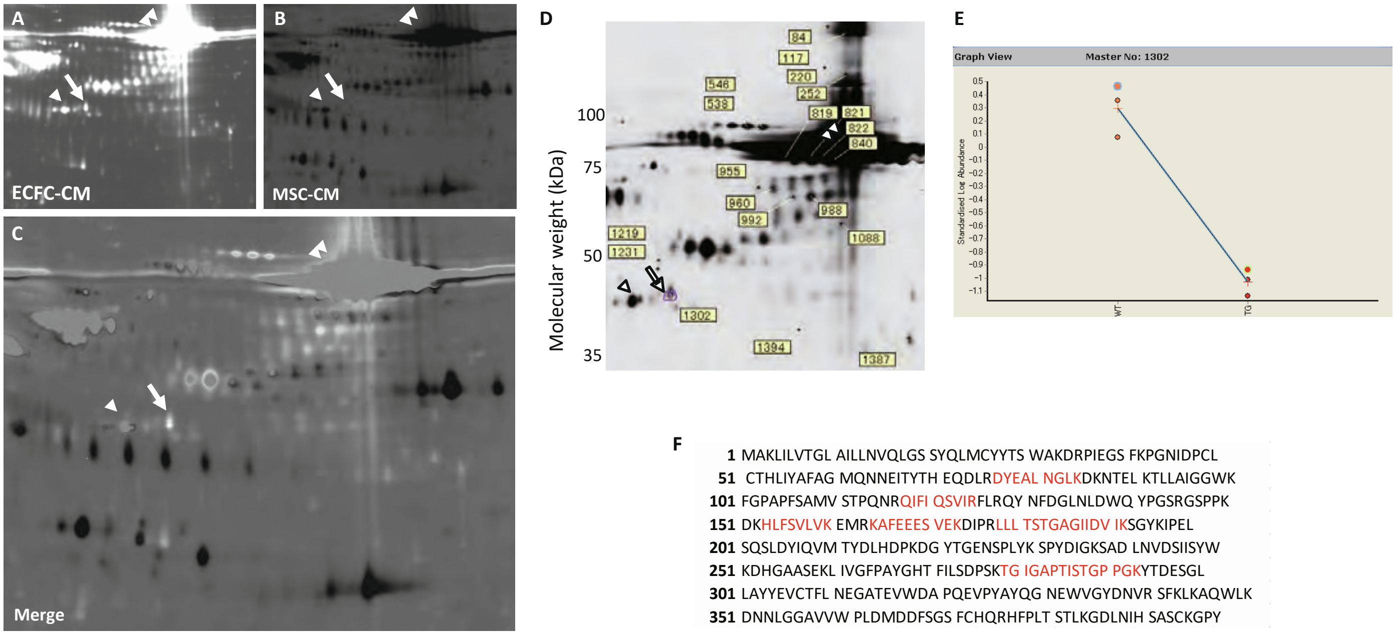

Figure S3. ECFC-CM-specific spots in 2D-DIGE (related to Figures 2D-2G). (A-C) Gray-scale images of 2D-DIGE showing spots from ECFC-CM (white in A and C) and MSC-CM (black in B and C). Arrow, the most ECFC-CM-specific spot; arrowheads and double arrowheads, nonspecific spots of β-actin and transferrin, respectively. (D) Spots of ECFC-CM for which the protein abundance ratio (ECFC-CM/MSC-CM) was > 4 (3 independent experiments, $p < 0.01$). Numbers correspond to Master No. in Figure 2G. (E) The spot most specific to ECFC-CM exposure (No. 1302) showing 22.3-fold more abundant than the corresponding spot after MSC-CM exposure ($p = 0.0005$). (F) Matched peptides of spot 1302 with the protein database search. The eluate of spot 1302 was processed for nanoLC-MS/MS and matched with seven peptides (red) from CHIL3 (sequence coverage = 16% of 398 amino acids, protein score = 370) in Swiss-Prot and NCBInr.

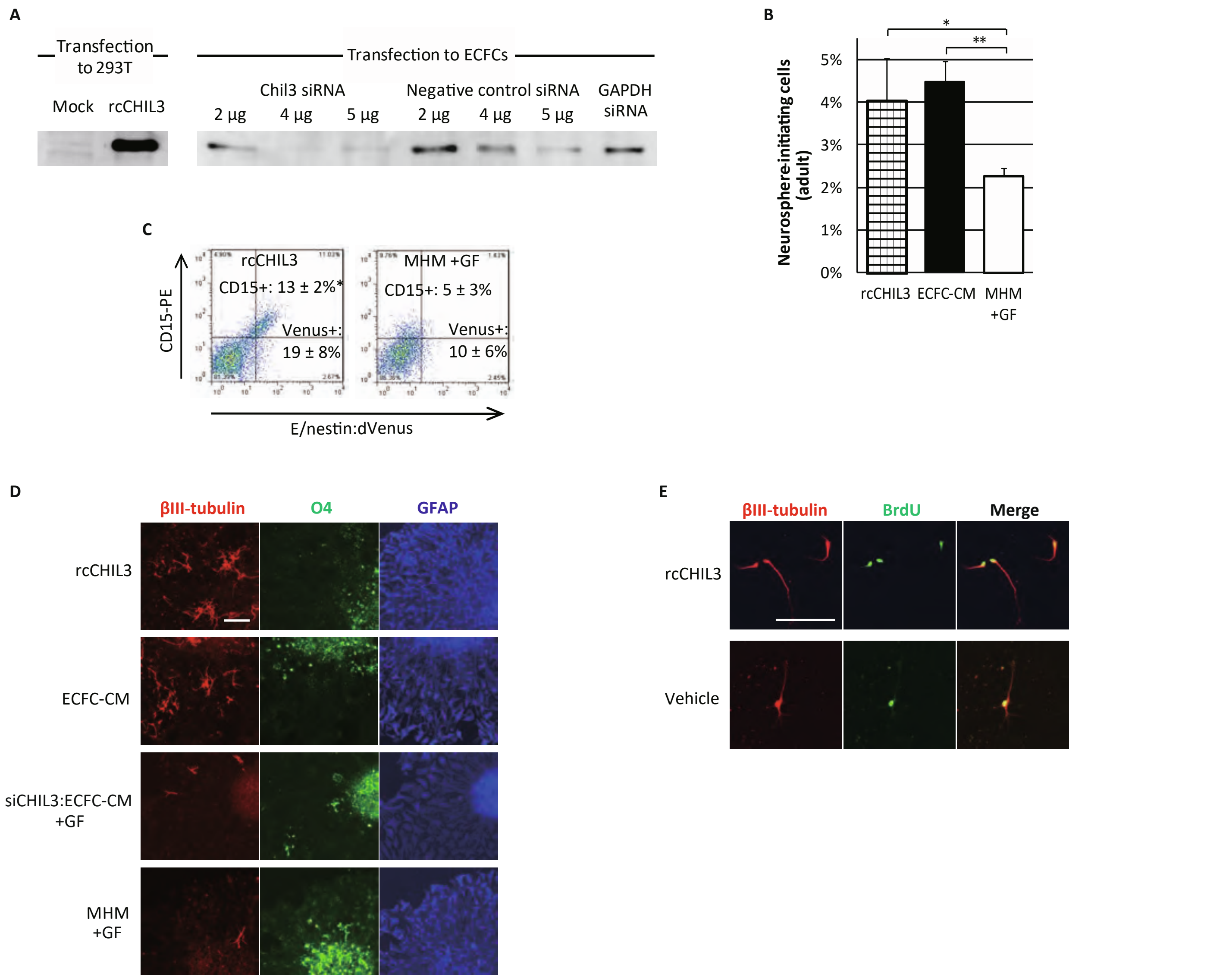

Figure S4. The neurosphere assay, flow cytometry of NSC markers, differentiation of neurospheres, and *ex vivo* differentiation of V-SVZ cells showing that rcCHIL3 promotes NSC self-renewal and neurogenesis (related to Figures 3A-3F). (A) Immunoblotting of rcCHIL3 and *Chil3* siRNA using CHIL3 antibody. Recombinant Venus protein was made as a mock experiment. siRNA that had no homology to any known mammalian gene was used as a negative control. CHIL3 was effectively depleted in ECFC-CM with *Chil3* siRNA of 4 or 5 μg/well. (B) Adult neurosphere assay with rcCHIL3, ECFC-CM, or MHM + GF. Data are mean ± SEM, *$p < 0.05$, **$p < 0.01$. 5 or 10 independent experiments. (C) Plots of flow cytometry showing expression levels of CD15 and Venus in the neurosphere cells treated with rcCHIL3 or MHM + GF. E/nestin:dVenus, a destabilized fluorescent protein Venus under the control of *Nes* second intronic enhancer. *$p < 0.05$, t-test versus MHM + GF controls. (D) Triple labeling immunocytochemistry after differentiation of neurospheres treated with rcCHIL3, ECFC-CM, siCHIL3:ECFC-CM+GF, or MHM + GF. βIII-tubulin (neurons, red), O4 (oligodendrocytes, green), and GFAP (astrocytes, blue). (E) Double labeling images of SVZ NSCs after *ex vivo* differentiation. βIII-tubulin (neurons, red) and BrdU (green). Adult mouse SVZ cells were dissected after 7 days of ventricular infusion with rcCHIL3 or vehicle, and cultured to differentiate with BrdU. Scale bar, 100 μm.

Figure S5

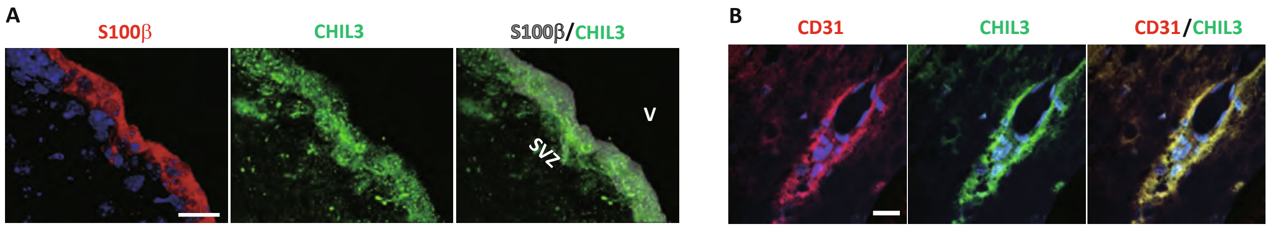

Figure S5. CHIL3 expression of S100β-positive ependymal cells (related to Figure 4J), and vascular cells in the brain after injury (related to Figure 5C). (A) The ventricular ependyma. (B) The injury site of the mouse brain after cold injury. Nuclei were stained with DAPI (blue). V, ventricle. Scale bars, 20 μm (A and B).

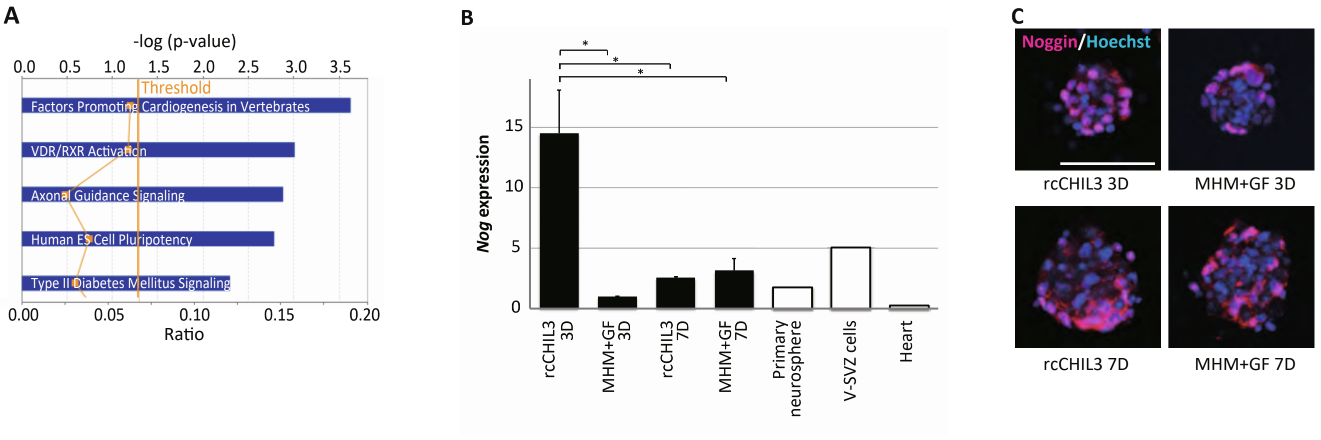

Figure S6. CHIL3-induced canonical pathways and noggin expression in NSCs, related to Figures 6A and 6D. (A) Canonical pathways of the genes classified into Cluster 1. Blue bars, p-value (-Log) calculated with Fisher' s exact test; orange line, threshold of $p = 0.05$; orange squares, the ratio of list genes found in each pathway over the total number of genes in that pathway. (B) qRT-PCR of *Nog* gene expression. Expression levels are expressed as relative ratios, with the expression level at MHM+GF 3D set as 1. 2 independent experiments for each. Data are mean ± SEM. $^*p < 0.05$. Samples of primary neurospheres, V-SVZ cells, and heart muscle cells were simultaneously examined as reference data. n = 1 each. (C) Immunocytochemistry of Noggin protein. CHIL3-treated NSCs highly express Noggin protein (red) as compared with MHM+GF-treated neurospheres at 3 DIV. At 7 DIV, Noggin expression illustrates cytoplasmic or intercellular distribution in neurospheres. Nuclei, blue. Scale bar, 20 μm.

Figure S7

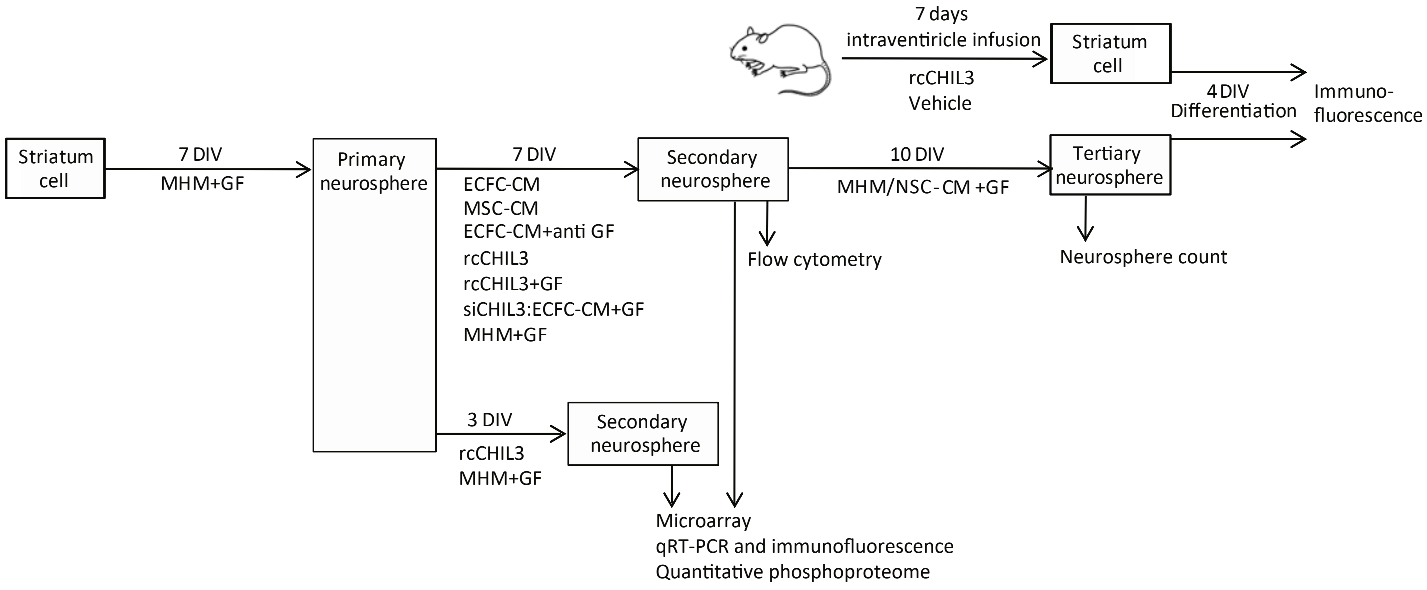

Figure S7. Schematic of cell culture protocol (related to Figures 2A-C, 3A-F, 5, and 6). Since dissected striatum cells are heterogenous, we used primary neurospheres and obtained secondary neurospheres that were treated with distinct media supplemented with test agents. To count neurosphere-initiating cells, which indicate the relative frequency of NSC self-renewal, we dissociated secondary neurospheres, cultured at clonal density with the NSC culture condition, and counted the number of tertiary neurospheres that were generated from the neurosphere-initiating cells in the test media-treated secondary neurosphere. To assess NSC differentiation, since neural stem/progenitor cells migrate away from the niche environment, differentiation culture media were not supplemented with test agents on the assumption as a component of the niche.

Supplementary Dataset 1. Genes and expression values of microarray data and results of cluster analysis (related to Figures 6A, 6D, and 6F). NSCs cultured with rcCHIL3 or control medium (MHM + GF) for 3 days (3D) or 7 days (7D) were processed for microarray analysis. Expression values are expressed as relative signal intensity after global normalization has been performed so that the average signal intensity of all probe sets is equal to 100. Signal detection of gene expression is statistically denoted as present (P), medium (M), or absent (A) by the MAS5 algorithm. Ratios < 1 are expressed as a reciprocal with a minus sign. The reproducibility of the gene expression ratio between two groups was calculated and expressed as ±4 (consistent reproducibility between the two groups when four pairs (2 independent experiments for each group) of the gene expression values are compared) through 0 (no significant difference in gene expression between those two groups).

Supplementary Dataset 2. Phosphorylated proteins, sites, and values of phosphoproteome data and results of cluster analysis (related to Figures 6B, 6C, 6E, and 6G). NSCs cultured with rcCHIL3 or control medium (MHM + GF) for days (3D) or 7 days (7D) were processed for quantitative phosphoproteome analysis. Peak areas of each phosphopeptide were normalized across samples treated with MHM + GF 3D, MHM + GF 7D, rcCHIL3 3D, and rcCHIL3 7D. Ratios are expressed in log2. 2 independent experiments for each group. Analyses were duplicated for each sample.

SUPPLEMENTAL EXPERIMENTAL PROCEDURES

ECFC, mature EC, EC line, MSC, and neurosphere culture

Adherent culture for ECFCs and mature ECs was established on fibronectin-coated 6-well plates (#140675, Nunc, Roskilde, Denmark). Following culture media were used: for ECFCs, endothelial basal medium supplemented with 5% fetal bovine serum (FBS), VEGF, FGF-2, R^3-IGF-1, EGF, hydrocortisone, ascorbic acid, and gentamicin/amphotericin-B (EGM-2-MV Bullet KitCC-3202, Lonza, Walkersville, MD); for committed mature ECs, endothelial maturation medium composed of endothelial basal medium with the supplements listed above but without FGF-2, R^3-IGF-1, and EGF. ECFCs were positive for Ki67, uptake of DiI-Ac-LDL, and rarely positive for von Willebrand factor. both ECFCs and mature ECs were positive for CD31 and VE-cadherin.

Cells of a mouse brain endothelioma cell line were maintained in Dulbecco's Modified Eagle Medium (#12699, Gibco Life Technologies, Carlsbad, CA) supplemented with 10% FBS and 1% penicillin G (10,000 U/mL)-streptomycin sulfate (10,000 mg/mL). The medium was renewed every 3-4 days. At the first passage, 3×10^6 cells were seeded on 6-well plates (9.6 cm^2 per well) with 3 mL of medium and cultured for 4 DIV.

For MSC culture, femurs and tibias of adult mice were dissected free of attached muscles, crushed, and suspended in αMEM (#11900, Gibco) supplemented with 10% FBS and 1% penicillin-streptomycin. Cell suspensions were filtered through a 70-μm filter. Cells (5×10^6 cells/mL) were cultured on non-coated 6-well plates with 3 mL of medium. At 24 h after plating, adherent cells were washed to remove non-adherent cells. The medium was changed weekly, and cells were nearly confluent at 21 DIV.

For neurosphere culture, the striata of embryonal day 14 or adult mouse forebrain were dissected and collected into phosphate-buffered saline (PBS) containing 0.6% glucose. Embryonal striata were mechanically triturated. Adult striata were incubated with trypsin solution for 15 min at 37 °C, triturated, and trypsin inhibitor solution was added. Dissociated cells (2×10^5 cell/mL for embryonic mice, 5000 cells/mL for adult mice) were cultured with serum-free neurosphere culture medium (MHM) supplemented with recombinant human EGF (20 ng/mL) and recombinant human FGF-2 (20 ng/mL) for 7 DIV, and formed floating primary neurospheres. MHM is composed of DMEM-F12 (1:1), glucose (0.6%), glutamine (2 mM), sodium bicarbonate (13.4 mM), HEPES (5 mM), insulin (25 mg/mL), transferrin (100 mg/mL), progesterone (20 nM), sodium selenate (30 nM), and putrescine (60 nM). Neurospheres were mechanically dissociated, clonally (10 cells/μL) cultured with distinct media and formed secondary neurospheres (Figure S7). The following neutralizing antibodies and concentrations were used for the neutralizing experiment (ECFC-CM + anti GF): mouse monoclonal anti-FGF-2 (10 μg/mL; 05-117, Millipore, Billerica, MA) and rabbit polyclonal anti-EGF (20 μg/mL; 06-102, Millipore). For the neurosphere assay, secondary neurospheres were mechanically dissociated into single cells, clonally plated with a 1:1 cocktail of MHM and primary neurosphere culture-conditioned medium supplemented with EGF and FGF-2 (MHM/NSC-CM + GF), and cultured, followed by generation of tertiary neurospheres (Figure S7). The percentage of neurosphere-initiating cells in the population of secondary neurospheres was calculated as 100 multiplied by the number of tertiary neurospheres (diameter > 50 μm) divided by the number of plated cells. Neurosphere counts were performed in a blinded fashion by the same observer.

Tertiary neurospheres or striatum cells from adult mice were allowed to differentiate into the CNS lineage (Figure S7). Neurospheres (one neurosphere/well) or dissociated striatum cells (1×10^4 cells/mL) were plated onto poly-L-ornithine- and fibronectin-coated chamber slides (#5732-008, Iwaki, Chiba, Japan) with MHM plus 1% FBS. Plated neurospheres were then gently triturated several times. Cells were cultured and fixed for immunocytochemistry at 4 DIV. To assess cell proliferation, BrdU (1 μM) was added to the medium.

Immunostaining

A cryostat (FINETEC CM3050S, Leica, Wetzlar, Germany) or a vibratome (VT1200S, Leica, Heidelberg, Germany) were used for coronal sections (14-μm or 50-μm, respectively) of adult

mouse brains. Postnatal days 5 or 16 or adult mouse eyes were isolated for whole-mount samples as previously described (Kubota et al., 2009). Vibratome sections were sequentially preincubated with ice-cold acetone and 1% H_2O_2 in PBS. Whole-mount samples were stored in methanol at -20 °C and rehydrated for staining.

The following primary antibodies and dilutions were used: sheep anti-BrdU (1:100; 20-BS17, Fitzgerald Industries International, Inc., Concord, MA), mouse monoclonal anti-mouse βIII-tubulin (1:1000; T8660, Sigma-Aldrich, St. Louis, MO), mouse monoclonal anti-O4 (1:1000; MAB345, Millipore), mouse monoclonal anti-GFAP (1:100; G3893, Sigma-Aldrich), polyclonal rabbit anti-GFAP (1:2000; Z0334, Dako, Carpinteria, CA), rat monoclonal anti-mouse YM1/Chitinase 3-like 3 (1:50 for immunostaining, 1:500 for immunoblotting, 1:100 for immuno-electron microscopy; MAB2446, R&D Systems, Inc., Minneapolis, MN), goat anti-mouse YM1/Chitinase 3-like 3 (1:1000; AF2446, R&D), rat anti-mouse CD31 (1:10; 550274, BD Biosciences, San Jose, CA), hamster anti-CD31 (1:1000; 2H8, Chemicon, Temecula, CA), goat anti-VE-cadherin (1:10; sc-6458, Santa Cruz Biotechnology, Inc., Santa Cruz, CA), goat anti-mouse Noggin (1:10; AF719, R&D), rabbit anti-Aquaporin 4 (1:200; AB3594, Millipore), mouse monoclonal anti-S-100 β-subunit (1:500; S2532, Sigma-Aldrich), and anti-Doublecortin (1:100; sc-8066, Santa Cruz Biotechnology, Inc.). GFP was not stained with an anti-GFP antibody for sections; crude GFP expression was observed under a fluorescence microscope. Anti-GFP (1:500; Alexa488-conjugated; Molecular Probes, Eugene, OR) was used only for whole-mount samples.

The following signal amplifications were applied for CD31 and VE-cadherin: horseradish peroxidase-conjugated secondary antibody (1:500; Jackson Laboratory, West Grove, PA) and Tyramide Signal Amplification (Renaissance TSA fluorescence system, NEL701-705 (green) or NEL702-705 (red), Perkin Elmer, Waltham, MA). Nuclei were stained with Hoechst 33258 (94403, Sigma-Aldrich) or 4',6-diamidino-2-phenylindole (DAPI, Molecular Probes). Controls lacking the primary antibody were processed in parallel for all histochemistry.

After staining, cells and sections were observed with a fluorescence microscope (BX-61, Olympus, Tokyo, Japan; or AxiCom, Zeiss Deutschland, Oberkochen, Germany) or a confocal laser microscope (LSM510 META, Zeiss; LSM5 Pascal, Zeiss; or FV1000, Olympus). Florescence images were prepared from data files with AxioVision 4 software or Zeiss Image Browser Ver. 3.5 software, and with Adobe Photoshop Ver. 12.1 software (San Jose, CA). Linear adjustments to brightness or contrast were applied to the entire image when necessary. The numbers of βIII-tubulin-, O4-, GFAP-, and BrdU-positive cells were counted in five non-overlapping visual fields at a magnification of 400×. Cell counts were performed in a blinded fashion by the same observer. The number of BrdU- and Doublecortin-positive cells in the right V-SVZ (the side of the intraventricle infusion) of eight serial in every tenth section was counted and summed in a blinded fashion by the same observer. BrdU-positive cells in the granule cell layer of the olfactory bulb (both injected side and contralateral side) were counted with the Dynamic Cell Count software under a fluorescence microscope (BZ-9000, Keyence, Osaka, Japan). A region of interest was set manually for the granule cell layer. Counts of three in every tenth section were summed.

Flow cytometry

Single-cell suspensions of secondary neurospheres were processed for flow cytometry. Cells were stained with fluorescein isothiocyanate anti-human CD15 (555401, BD Biosciences) or phycoerythrin anti-human CD15 (55402, BD Biosciences). PI (2 μg/mL) was added to discriminate dead cells from live cells. Cell suspensions were analyzed with a FACS Vantage (Becton Dickinson). Cells obtained from E/nestin:dVenus transgenic mouse embryos were assayed for crude Venus expression. The proportions of CD15- and Venus-positive cells among all live cells were obtained by analyzing the cumulative data from each animal.

ELISA
The following ELISA kits were used according to the manufacturer's instructions: Quantikine Human FGF Basic (DFB50), Quantikine Mouse EGF (MEG00), Quantikine Mouse VEGF (MMV00), Quantikine Human BDNF (DBD00), and Quantikine Mouse Chitinase 3-like 3/ECF-L (MC3L30, all kits from R&D).

For CSF collection, mice were anesthetized with an inhalant anesthetic and placed on a stereotactic frame (SR-5, Narishige, Tokyo, Japan) in a position that flexed the neck. A midline incision on the neck was made, and then the skull of the posterior part and the upper cervical vertebrae were exposed. CSF was gently drawn up at the puncture at the dura mater of the cisterna magna. CSF obtained from one animal or a pooled sample from a few animals was processed for ELISA since the volume of CSF obtained was less than 50 µL per animal.

Supernates of the blood were collected as serum after coagulation and centrifugation. Brain cells were rinsed with PBS, chopped into 1-2 mm pieces, and cultured in Roswell Park Memorial Institute medium with 2 mM L-glutamine (11875-093, Gibco) supplemented with 10% FBS and penicillin-streptomycin. Culture supernates of brain cells were removed at 4 DIV and assayed.

Immuno-electron microscopy
Frozen sections were incubated with rat monoclonal anti-mouse CHIL3 primary antibody followed by incubation with biotin-conjugated donkey anti-rat secondary antibody (1:1000; 712-065, Jackson ImmunoResearch Laboratories, Inc., West Grove, PA) and with Alexa488- and nanogold-conjugated streptavidin (1:100; A-24926, Molecular Probes Invitrogen, Carlsbad, CA). After enhancement with Silver Enhancement Kit (HQ Silver, Catalog number 2012, Nanoprobes Inc., Yaphank, NY), sections were post-fixed, dehydrated, and embedded in Epon. Ultrathin sections were stained with uranyl acetate and lead citrate and observed under a transmission electron microscope (model 1230, JEOL, Tokyo, Japan). Images were taken with Digital Micrograph 3.3 (Gatan Inc., Pleasanton, CA).

Differential proteomics
ECFC-CM and MSC-CM were pretreated with Multiple Affinity Removal Spin Cartridges (Mouse 3, Agilent Technologies, Santa Clara, CA) according to the manufacturer's instructions to remove albumin, IgG, and transferrin. Proteins were extracted and processed. A pooled sample (1:1 weight mixture of ECFC-CM and MSC-CM), ECFC-CM, and MSC-CM were labeled with Cy2, Cy3, and Cy5 (GE Healthcare Biosciences, Uppsala, Sweden), respectively. Protein aliquots (200 µg) were first separated according to isoelectronic point with Multiphore II (GE Healthcare Biosciences) and Immobilized pH Gradient Strips (24 cm, pI3-10, GE Healthcare Biosciences). Sodium dodecyl sulfate polyacrylamide gel electrophoresis was applied to the second dimension, separating the proteins by mass, with an Ettan DALT II Electrophoresis System (GE Healthcare Biosciences). The gel images were acquired with Typhoon (GE Healthcare Biosciences) and processed for spot detection, background subtraction, normalization, quantitation of spots in images from a single gel, spot matching on multiple gels, and statistical analysis of changes in protein abundance with DeCyder 2D Differential Analysis Software (GE Healthcare Biosciences).

A 600-µg protein extract from ECFC-CM was loaded for two-dimensional electrophoresis. Spots on the gel were stained with Sypro Ruby Protein Gel Stain (#S12001, Molecular Probes Invitrogen). The gel was imaged with MasterImager (GE Healthcare Biosciences). Spots of interest were matched by DeCyder 2D Differential Analysis Software (GE Healthcare Biosciences), picked from the gel with an Ettan Spot Picker (GE Healthcare Biosciences), digested, and analyzed via nanoLC-MS/MS with the CapLC system (Waters, Milford, MA) and the Micromass Q-Tof Micro Mass Spectrometer (Waters) with MassLynx Software (Waters).

rcCHIL3
Plasmid DNA containing the sequence encoding *Chil3* was obtained from *Escherichia coli* DH10B TonA with the pDNR-LIB vector encoding the mouse *Chil3* cDNA (IRALp962M1053Q, Source

BioScience imaGenes, Berlin, Germany) with a QIAprep Spin Miniprep Kit (27104, QIAGEN Inc., Valencia, CA) and transferred into the pLP-CMV-Myc acceptor vector (631603, Clontech Laboratories, Inc., Mountain View, CA). DH5α cells were transfected with the acceptor vector and transformed by heat shock. Plasmid DNA was retrieved from the DH5α competent cells with a QIAprep Spin Miniprep Kit (27104, QIAGEN) and scaled up with the Qiaprep Spin Maxiprep Kit (10262, QIAGEN). Expression vector pCS2+ harboring the gene encoding Venus was used for mock experiments. HEK293T cells were cultured on poly-L-ornithine-coated 10-cm plastic dishes for 1 DIV. Then, a mixture of *Chil3* DNA (6 µg), Opti-MEM I Reduced-Serum Medium (800 µL, 31985-070, Gibco), and GeneJuice Transfection Reagent (15 µL, 70967, Novagen, Darmstadt, Germany) was added to 15 mL of culture medium in a 10-cm dish. The cells were cultured for another 1 DIV. The culture medium was then changed to MHM 1 day before obtaining rcCHIL3.

CHIL3 knock-down

ECFCs were established in a 6-well plate as described above. At 21 DIV, a siRNA complex of *Chil3* consisting of siRNA (4 µg/well), Opti-MEM I Reduced-Serum Medium (80 µL/well, 31985-070, Gibco), and X-tremeGENE siRNA Transfection Reagent (20 µL/well, 04 476 093 001, Roche Diagnostics, Mannheim, Germany) was added to the culture medium, and the cells were cultured for another 1 DIV. The culture medium was changed and the conditioned medium, siCHIL3: ECFC-CM, was then obtained. siRNA against the gene encoding glyceraldehyde 3-phosphate dehydrogenase (GAPDH; Silencer FAM, AM4650, Ambion Invitrogen) and negative control siRNA provided by the Sigma Genosys siRNA Service were used as controls.

Microarray analysis

Total RNA was isolated from rcCHIL3-treated or MHM + GF-treated secondary neurospheres with the Qiagen RNeasy Mini Kit (74104, QIAGEN, Hilden, Germany). DNA microarray analysis using Affymetrix Gene Chip technology was performed as described previously (Heishi et al., 2008; Heishi et al., 2006; Ishida et al., 2002; Matsui et al., 2012). Briefly, 100 ng of total RNA was used as a template for cDNA synthesis, and biotin-labeled cRNA was synthesized with the 3' IVT Express Kit (901228, Affymetrix, Santa Clara, CA). After generating the hybridization cocktails, hybridization to the DNA microarray (900496, GeneChip Mouse Genome 430 2.0 Array, Affymetrix) (Lockhart et al., 1996) and fluorescent labeling were performed. The microarrays were then scanned with a GeneChip Scanner 3000 7G System (Affymetrix). Data analysis was carried out using GeneChip Operating Software 1.04 (Affymetrix). Signal detection and quantification were performed using the MAS5 algorithm with default settings.

For the clustering analysis, signals were normalized, calculated, and visualized with Spotfire DecisionSite System 9.1.2 (TIBCO, Palo Alto, CA). Principal component analysis was carried out with Spotfire DecisionSite 9.1.2 using normalized data. Pathway analysis was performed with Ingenuity Pathway Analysis ver.14197757 (Ingenuity Systems, Redwood City, CA).

qRT-PCR

qRT-PCR for *Nog* was performed according to the manufacturer's instructions (QIAGEN). Total RNA was isolated from rcCHIL3-treated or MHM + GF-treated secondary neurospheres, primary neurospheres, V-SVZ cells, and heart muscle cells (from embryonal day 14 mouse embryos) with the miRNeasy Mini Kit (217004, QIAGEN), and was treated with an RNase-free DNase set (79254, Qiagen) to remove contaminating genomic DNA. We used the QuantiFast SYBR Green PCR Kit (204052, Qiagen) and the ABI StepONePlus instrument and software v2.1 (Applied Biosystems Life Technologies, Carlsbad, CA) for qRT-PCR with the following qPCR program: 95 °C for 5 min and 40 cycles of 95 °C for 10 s, 60 °C for 30 s, and 72 °C for 15 s. Melting-curve analysis was routinely used for each reaction. The gene encoding GAPDH was run in parallel as an internal control for each reaction set.

Quantitative phosphoproteome analysis

Proteins were extracted from 100,000 cells using 12 mM sodium deoxycholate and 12 mM sodium lauroyl sarcosinate, and digested with Lys-C and trypsin (Masuda et al., 2009). Phosphopeptides were enriched by aliphatic hydroxy acid-modified metal oxide chromatography with titania (Sugiyama et al., 2007) and analyzed by nanoLC-MS/MS using an LTQ-Orbitrap instrument (Thermo Fisher Scientific, Bremen, Germany). Peptides and proteins were identified using Mascot version 2.3 (Matrix Science, London, UK) with the Swiss-Prot database. Label-free quantitation was performed based on the peak areas of extracted ion chromatograms for identified phosphopeptides using Mass Navigator (Mitsui Knowledge Industry, Tokyo, Japan). Two independent experiments for each group. Analyses were duplicated for each sample.

For the clustering analysis, the peak area of each phosphopeptide was normalized across samples of the culture groups. The normalized peak areas and the peak area ratios were subjected to cluster analysis with Cluster version 2.11 (http://rana.lbl.gov/EisenSoftware.htm) (Eisen et al., 1998).

In vivo **intraventricle infusion**

Adult mice were anesthetized with an inhalant anesthetic and placed on a stereotactic frame (SR-5, Narishige). A cannula (0008851 ALZET Brain Infusion Kit 3, DURECT Co.) was implanted stereotaxically at the coordinates of anteroposterior = 0 mm, mediolateral = –1.2 mm, and dorsoventral = –2.3 mm relative to the bregma, and glued with a cyanoacrylate adhesive onto the skull. A mini-osmotic pump (0.5 µL/h, model 1007D ALZET micro-osmotic pump, DURECT Co., Cupertino, CA) was attached to the cannula and implanted subcutaneously into the dorsal flank. After surgery, mice were housed before sacrifice.

For the immunohistochemical preparations, mice were transcardially perfused with PBS, followed by 4% paraformaldehyde. Forebrains were removed and processed. For *ex vivo* culture, forebrains were aseptically removed and processed.

REFERENCES

Eisen, M.B., Spellman, P.T., Brown, P.O., and Botstein, D. (1998). Cluster analysis and display of genome-wide expression patterns. Proc Natl Acad Sci U S A *95*, 14863-14868.

Heishi, M., Hayashi, K., Ichihara, J., Ishikawa, H., Kawamura, T., Kanaoka, M., Taiji, M., and Kimura, T. (2008). Comparison of gene expression changes induced by biguanides in db/db mice liver. The Journal of toxicological sciences *33*, 339-347.

Heishi, M., Ichihara, J., Teramoto, R., Itakura, Y., Hayashi, K., Ishikawa, H., Gomi, H., Sakai, J., Kanaoka, M., Taiji, M.*, et al.* (2006). Global gene expression analysis in liver of obese diabetic db/db mice treated with metformin. Diabetologia *49*, 1647-1655.

Ishida, N., Hayashi, K., Hoshijima, M., Ogawa, T., Koga, S., Miyatake, Y., Kumegawa, M., Kimura, T., and Takeya, T. (2002). Large scale gene expression analysis of osteoclastogenesis in vitro and elucidation of NFAT2 as a key regulator. J Biol Chem *277*, 41147-41156.

Kubota, Y., Takubo, K., Shimizu, T., Ohno, H., Kishi, K., Shibuya, M., Saya, H., and Suda, T. (2009). M-CSF inhibition selectively targets pathological angiogenesis and lymphangiogenesis. The Journal of experimental medicine *206*, 1089-1102.

Lockhart, D.J., Dong, H., Byrne, M.C., Follettie, M.T., Gallo, M.V., Chee, M.S., Mittmann, M., Wang, C., Kobayashi, M., Horton, H.*, et al.* (1996). Expression monitoring by hybridization to high-density oligonucleotide arrays. Nature biotechnology *14*, 1675-1680.

Masuda, T., Saito, N., Tomita, M., and Ishihama, Y. (2009). Unbiased quantitation of Escherichia coli membrane proteome using phase transfer surfactants. Mol Cell Proteomics *8*, 2770-2777.

Matsui, T., Takano, M., Yoshida, K., Ono, S., Fujisaki, C., Matsuzaki, Y., Toyama, Y., Nakamura, M., Okano, H., and Akamatsu, W. (2012). Neural stem cells directly differentiated from partially reprogrammed fibroblasts rapidly acquire gliogenic competency. Stem Cells *30*, 1109-1119.

Sugiyama, N., Masuda, T., Shinoda, K., Nakamura, A., Tomita, M., and Ishihama, Y. (2007). Phosphopeptide enrichment by aliphatic hydroxy acid-modified metal oxide chromatography for nano-LC-MS/MS in proteomics applications. Mol Cell Proteomics *6*, 1103-1109.
